# Discovery of Species-Specific Peptide Markers for Superseed Authentication Using Targeted LC-MS/MS Proteomics

**DOI:** 10.3390/molecules30142993

**Published:** 2025-07-16

**Authors:** Sorel Tchewonpi Sagu, Beatrice Schnepf, Peter Stenzel, Kapil Nichani, Alexander Erban, Joachim Kopka, Harshadrai M. Rawel, Andrea Henze

**Affiliations:** 1 Institute of Agricultural and Nutritional Sciences, Martin Luther University Halle-Wittenberg, Von-Danckelmann-Platz 2, 06120 Halle (Saale), Germany; andrea.henze@landw.uni-halle.de; 2 Institute of Nutritional Science, University of Potsdam, Arthur-Scheunert-Allee 114-116, 14558 Nuthetal, Germany; bea.schnepf@gmail.com (B.S.); kapil.nichani@uni-potsdam.de (K.N.); hmrawel@yahoo.de (H.M.R.); 3 Max-Planck-Institut of Molecular Plant Physiology, Potsdam Science Park, Am Mühlenberg 1, 14476 Potsdam, Germany; p.stenzel@uke.de (P.S.); erban@mpimp-golm.mpg.de (A.E.); kopka@mpimp-golm.mpg.de (J.K.)

**Keywords:** superfoods, superseeds, authentication, protein extraction, marker peptide, targeted mass spectrometry

## Abstract

The increasing popularity of “superseeds” such as flax, sesame, amaranth and quinoa as functional foods raises the need for robust analytical methods for authentication purposes. In this work, a standardized workflow for the extraction, characterization and identification of unique peptides that may be used as markers to distinguish superseed species was investigated. Ammonium bicarbonate/urea (Ambi/urea) extraction, sodium dodecyl sulfate (SDS) buffer and trichloroacetic acid (TCA) precipitation were initially implemented and, based on the level and composition of the extracted proteins, the SDS buffer protocol was selected. Electrophoresis analysis revealed consistent protein profiles between biological replicates from each of the eleven seed species, confirming the reproducibility of the SDS buffer protocol. Targeted mass spectrometry successfully identified species-specific peptide markers for six of eleven superseeds investigated, including peptides from conlinins in flaxseed (WVQQAK), 11S globulins in sesame (LVYIER), oleosin in quinoa (DVGQTIESK), agglutin-like lectins in amaranth (CAGVSVIR), as well as cupin-like proteins in poppy seeds (INIVNSQK) and edestins in hemp seeds (FLQLSAER). Moreover, proteome cross-analysis allowed us to disqualify the isomeric peptide LTALEPTNR from 11S globulins present in amaranth and quinoa. However, no reliable markers were identified for chia, canihua, basil, black cumin, and psyllium seeds under current conditions. While this targeted proteomics approach shows promise for superseed authentication, comprehensive method validation and alternative strategies for marker-deficient species are required before routine implementation.

## 1. Introduction

In recent years, dietary habits have undergone a marked transformation, with growing consumer interest in health-promoting and organic foods. Among these so-called “superfoods”, the nutrient-rich plant-based products have gained substantial popularity. Between 2011 and 2015, global product launches featuring superfoods, superfruits, and ancient grains increased by over 200% [1]. In particular, edible superseeds are attracting increasing attention, not only for their nutritional and therapeutic properties, but also for their rich composition of macronutrients and micronutrients, including proteins, dietary fibers, lipids (notably unsaturated fatty acids), and various vitamins and minerals [2,3,4,5].

Several examples highlight this nutritional diversity: poppy and black cumin seeds contain 21–27% protein and 33–40% fat, mainly composed of linoleic and oleic acids [4,6,7]. Flaxseed is valued for its high content of α-linolenic acid (omega-3), soluble fiber, and proteins [8,9,10]. Hemp seed, traditionally consumed in human diets, provides 25–32% protein and up to 35% fat and is particularly rich in polyunsaturated fatty acids [11,12]. Seeds from the Lamiaceae family, such as chia, basil, sesame, and psyllium, are also known for their high nutritional density, offering omega-3 fatty acids, high-quality proteins, dietary fiber, polyphenols, and essential micronutrients [2,13]. Sesame seeds contain 40–65% fat and 19–35% protein [14,15,16], while psyllium is particularly appreciated for its high dietary fiber content [17]. Finally, pseudo cereals such as quinoa, amaranth, and canihua are gluten-free and exhibit well-balanced amino acid profiles, making them attractive alternatives for individuals with celiac disease [18,19,20,21].

However, with their growing attractiveness and ever-increasing market value, superseeds as the most important and high-valued plant-based foods are vulnerable to food fraud and adulteration. These adulterations generally involve the replacement of high-value, high-quality ingredients with equivalents of inferior value and quality [22,23,24,25,26]. To prevent these frauds, a variety of analytical techniques are generally developed and applied for food authentication, including the PCR Method, ELISA, Raman spectrometer, ICP-MS, SNIF-NMR, MALDI-TOF-MS, and liquid and gas mass spectrometry [26,27,28,29,30]. Initiatives have also been implemented for superseeds. Machine learning algorithms such as random forest were employed to discover untargeted metabolic markers distinguishing seeds like chia from flax and sesame [31]. Targeted analysis confirmed the presence of seed-specific polyphenols [32] and more recently, metabolomics approaches based on NMR spectroscopy identified potential authenticity markers in flax, sesame, and chia, including polyphenols and cyanogenic glycosides [3]. However, if metabolic profiling has been widely used, its limitations in species discrimination and stability under processing conditions highlight the need for alternative or complementary methods. Compared to metabolomics-based approaches, proteomics offers a complementary and often more robust strategy for food authentication since the proteomes are inherently species-specific, more stable than many small metabolites, and directly linked to genetic expression [33,34]. This makes it possible to identify unique protein or peptide markers that remain reliable across varying environmental and processing conditions.

Despite these advantages, protein-based authentication of superseeds remains largely unexplored. A survey of the UniProt database (as of January 2024) shows that most superseed proteins remain unreviewed, and only a few have been fully characterized, limiting the availability of reliable protein biomarkers. Moreover, while certain seeds (e.g., sesame, chia, flax, quinoa) have been the subject of proteomic studies, others such as basil, psyllium, and amaranth seeds remain poorly documented. Peptides derived from tryptic digestion of storage proteins primarily 11–12S and 7–8S globulins, as well as 2S albumins, which together represent more than 70% of total seed proteins, are promising candidates for authentication. These proteins are known to vary with genotype, geographical origin, farming practices (organic vs. conventional), and processing conditions (e.g., freezing, heating, fermentation) [35,36], and can therefore be good candidates for the search for markers to enable the traceability and authentication of superseeds.

In light of the information above, this study aimed to develop a standardized workflow for the discovery of robust specific peptide markers that can be used for authentication of some selected superseeds based on targeted mass spectrometry analysis. To ensure reproducibility and analytical reliability, three different extraction protocols were initially investigated and their efficiency compared, including ammonium bicarbonate (Ambi/urea) extraction, sodium dodecylsulfate (SDS) buffer, and trichloroacetic acid (TCA) precipitation. The profiles of extracted proteins were then analyzed by sodium dodecyl sulfate polyacrylamide gel electrophoresis (SDS-PAGE) and further subjected to targeted mass spectrometry analysis to identify potential peptide markers. The selected peptide markers were subsequently monitored by multiple-reaction monitoring (MRM) in 81 seed samples from 11 superseed species, including flax, chia, sesame, amaranth, hemp, quinoa, canihua, poppy, basil, black cumin, and psyllium seeds.

## 2. Results

### 2.1. Initial Sample Assessment

#### 2.1.1. Crude Protein

Prior to targeted analysis, one representative sample per seed species was selected and analyzed for total protein content. The Kjeldahl method was used to determine total nitrogen, which was subsequently converted to protein content. Among the samples, flaxseed S43 (20.3 g/100 g dry mass), sesame S62 (25.2 g/100 g dry mass), hemp S201 (23.3 g/100 g dry mass), and poppy seed S500 (21.9 g/100 g dry mass) exhibited relatively high protein levels. In contrast, amaranth S101 and quinoa S302 showed lower protein contents, with 14.9 and 13.0 g/100 g dry mass, respectively.

The protein content of flaxseed S43 (20.3% dry mass) aligned with literature values, which typically ranged from 18.3% to 22% dry mass [9,37]. Similarly, sesame S62 (25.2% dry mass) was situated at the upper end of the reported range of 17–25% dry mass [16,38]. Amaranth S101 (14.9% dry mass) also fell within its expected range of 13.6–15% dry mass [39]. Hemp S201 exhibited a slightly lower protein content (23.3%) compared to the literature (25–31.6% dry mass) [2]. The protein content of quinoa S302 (13.0% dry mass) was within the lower end of the wide range reported in the literature (10–22% dry mass) [40]. Lastly, the poppy seed sample S500 yielded a protein content of 21.9%, also consistent with the reported range of 21–27% dry mass [41,42].

#### 2.1.2. Fat Content

To prevent interference with subsequent analyses, seed lipids were removed prior to protein extraction. For this purpose, ground seed samples were extracted twice with hexane; fat content was calculated based on the weight difference before and after defatting, and the results, expressed in percentages, are shown in Figures S1 and S2 (Supplementary Materials). These figures highlight considerable variability in fat content within each seed type, reflecting differences that may stem from varietal genetics, geographical origin, or processing conditions. While quinoa and amaranth consistently exhibited low fat levels below 6% (Figure S2C,E), chia, basil, and hemp seeds displayed moderate fat contents ranging from 14% to 22%, and black cumin, flaxseed, poppy seed, and sesame demonstrated the highest overall fat levels, between 26% and 37%. On average, the measured fat contents were lower than those typically reported in the literature; 42–48 g/100 g for flaxseed and 30–33 g/100 g for chia [43], 17.7–25 g/100 g for sesame, 7.0 g/100 g for amaranth, and 35–48.8 g/100 g for hemp. The reported values for quinoa are around 6.1 g/100 g, for canihua 6–8 g/100 g, for poppy seeds 33–41.6 g/100 g, for basil 9.7–33 g/100 g, and for black cumin 35–41 g/100 g. This observed variability is critical when standardizing protein extraction protocols, as lipid content can influence both extraction efficiency and matrix behavior.

### 2.2. Protein Extraction

**Figure 1:**
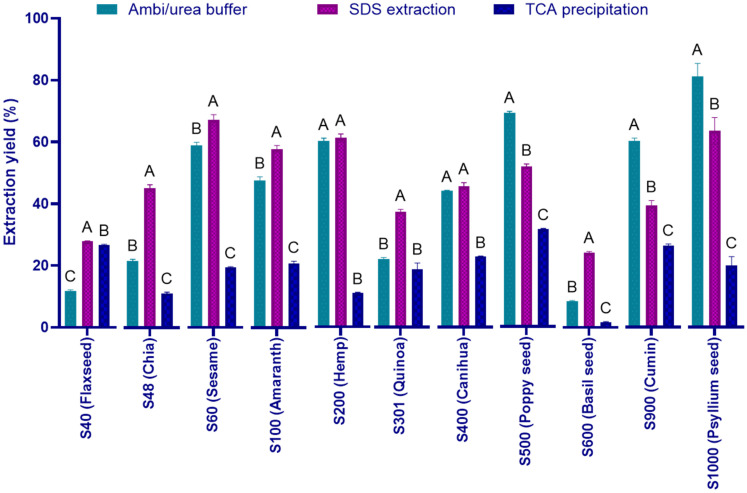
Protein extraction yields in comparison to total protein content determined by Kjehldahl (in %). Extraction was performed using Ambi/urea, SDS, and TCA-based protocols. One representative sample was analyzed for each of the eleven superfoods: S40 flaxseed, S48 chia, S60 sesame, S100 amaranth, S200 hemp, S301 quinoa, S400 canihua, S500 poppy seed, S600 basil seed, S900 cumin, and S1000 psyllium seed. Different letters indicate statistically significant differences between extraction methods (*p* < 0.05).

Figure 1 presents the extraction efficiencies obtained using three distinct methods, (1) Ambi/urea buffer, (2) SDS-based extraction, and (3) TCA precipitation, applied to a selected sample of each superseed type. The efficiency of protein extraction varied substantially depending on both the seed matrix and the method employed. Overall, the Ambi/urea buffer method provided the highest protein recoveries across most seed types, with yields exceeding 80% in psyllium seed (S1000), and surpassing 60% in poppy seed (S500), black cumin (S900), and sesame (S60). These results indicate a broad compatibility of the Ambi/urea approach with diverse seed compositions, likely owing to its strong solubilizing capacity for various protein classes. Nevertheless, the Ambi/urea method also exhibited clear limitations in several cases. In specific samples such as flaxseed (S40), chia (S48) and basil (S600), the extraction mixture developed a viscous, gel-like consistency during the procedure, rendering pipetting of the supernatant difficult and drastically reducing the recoverable volume. This phenomenon likely results from the presence of polysaccharides capable of gel formation even at low concentrations, as is well documented for chia seed hydrocolloids [44] and flaxseed mucilage [45,46]. To mitigate this problem, we experimented with extraction by reducing the quantities of initially weighed defatted samples from 100 mg to 50 mg, while maintaining the extraction buffer at 1 mL. This approach further reduced the gelatinous consistency and improved extraction yields.

SDS-based extraction also showed strong performance, particularly with flax (S40) chia (S48), sesame (S60), amaranth (S100), basil (S600) and hemp (S200), yielding protein quantities comparable to those obtained via the Ambi/urea method (Figure 1). This suggests that SDS is especially effective at solubilizing hydrophobic proteins, which are abundant in oil-rich seeds. In contrast, TCA precipitation consistently produced the lowest yields across all seed types, often below 25%. Notably, basil seed (S600) exhibited low extraction efficiency (<5%) across all types. Although commonly used for protein precipitation and cleanup, the strong denaturing conditions and limited solubilization associated with TCA likely contributed to its reduced extraction efficiency in this study. It should be noted that the overall efficiency of the TCA precipitation method observed may also be influenced by the specific extraction buffer used prior to the precipitation step. Although our primary focus was to compare extraction protocols under consistent conditions, the possibility that different pre-TCA buffers could impact protein solubilization and subsequent recovery cannot be excluded [47]. This factor is therefore acknowledged as a potential source of variability in protein yield that may be considered in future studies.

Statistical analysis revealed significant differences (*p* < 0.05) between the extraction methods within each seed type, as denoted by the letter groupings in Figure 1. These findings underscore the critical importance of method selection for optimizing protein extraction, and they demonstrate the broad applicability of both the Ambi/urea and SDS methods in superseed matrices. In fact, SDS and urea are commonly used in protein extraction processes for their denaturing and solubilizing properties. While urea is known as a chaotropic agent which disrupts hydrogen bonds and hydrophobic interactions within proteins, leading to their denaturation and solubilization, SDS is a powerful anionic detergent which denatures proteins by coating them with negatively charged molecules, causing them to unfold and become soluble. However, due to the practical limitations discussed above, the Ambi/urea method was excluded from further analyses. The SDS-based method was thus retained for its robustness and compatibility across all tested species.

**Figure 2:**
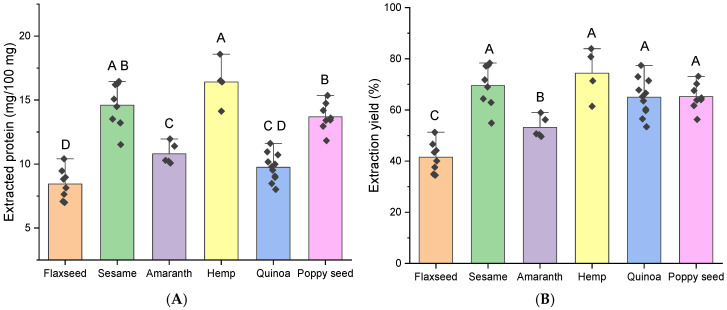
Amount of (**A**) extracted protein and (**B**) protein extraction yield using the standardized final SDS extraction method for all samples from flaxseed, sesame, amaranth, hemp, quinoa, and poppy seed. Letters A, B, C and D express significant differences (*p* < 0.05) according to a one-way ANOVA.

A modified SDS protocol incorporating reducing (TCEP) and alkylating (IAA) treatments was also tested but did not yield further improvements in extraction efficiency (Figure S4, Supplementary Materials). As an example, values of 5.71 ± 0.14; 12.82 ± 0.05; and 9.12 ± 0.29 (SDS-buffer extraction) and 6.15 ± 0.10; 13.40 ± 0.13; and 8.91 ± 0.19 (SDS-buffer extraction including reduction and alkylation) were obtained for the S40 flaxseed, S60 sesame, and S900 black cumin samples, respectively. Furthermore, a pairwise comparison analysis indicated that these results were not significantly different from each other (*p* > 0.05). Consequently, the original SDS protocol was selected as the standardized protein extraction method and applied to all seed samples; the results are presented in Figure 2. On average, amounts of extracted protein of 8, 15, 11, 17, 9, and 13 mg per 100 mg defatted sample were obtained for flaxseed, sesame, amaranth, hemp, quinoa, and poppy seed, respectively (Figure 2A). Corresponding extraction yields (Figure 2B) exceeded 50% for most samples across species, except for flaxseed samples, which consistently exhibited lower yields around 40%. This may be attributed to the high mucilage content, a key constituent of flaxseed, which likely hampers the efficiency of protein extraction [48,49].

### 2.3. Profile of the Extracted Proteins

**Figure 3:**
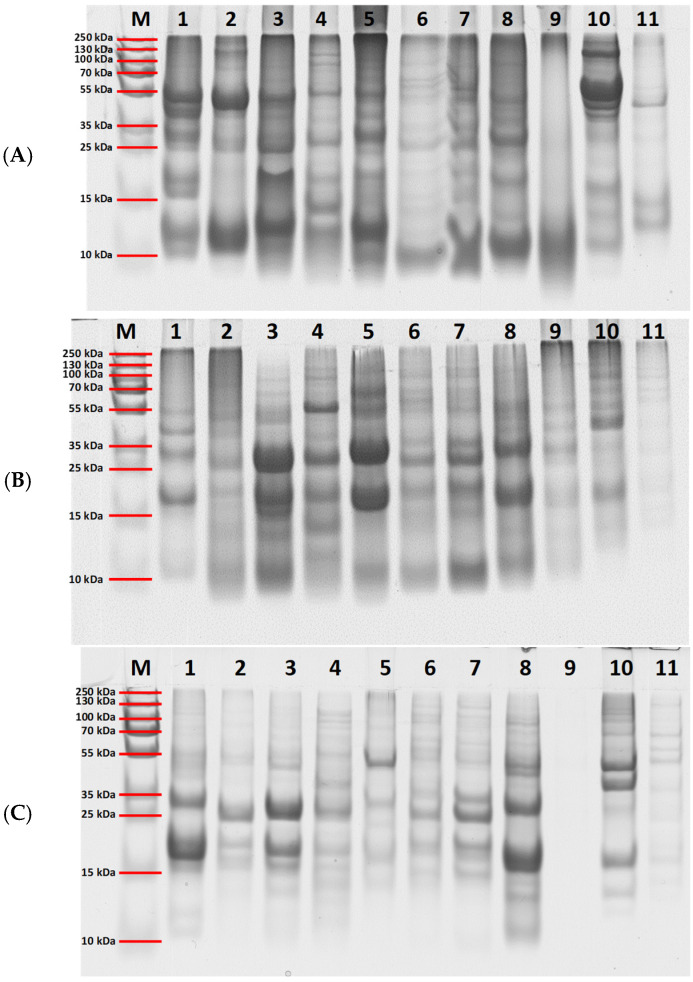
SDS-PAGE analysis of protein extracts obtained using three different methods: (**A**) Ambi/urea buffer extraction, (**B**) SDS extraction, and (**C**) TCA precipitation. The SDS-PAGE analyses were conducted with normalized protein amounts based on the Lowry assay, and ~25 µg proteins were loaded into each well. Lane M: molecular weight marker; lanes 1–11 correspond to the selected superfood samples: 1—S40 flaxseed, 2—S48 chia, 3—S60 sesame, 4—S100 amaranth, 5—S200 hemp, 6—S301 quinoa, 7—S400 canihua, 8—S500 poppy seed, 9—S600 basil seed, 10—S900 black cumin, 11—S1000 psyllium seed.

The protein profiles of eleven selected superseed samples extracted using Ambi/urea buffer, SDS buffer, and TCA precipitation were evaluated by SDS-PAGE, and the results are shown in Figure 3. The gel images reveal clear differences in protein band patterns and intensities across the extraction methods, reflecting variations in protein solubility and recovery efficiency, as discussed previously. The Ambi/urea (Figure 3A) and SDS-based extractions (Figure 3B) produced more distinct and diverse banding profiles than TCA precipitation (Figure 3C). Using the Ambi/urea buffer, distinct protein bands were visible in the 10–15 kDa range across all eleven seed species analyzed: flaxseed (1), sesame (2), chia (3), amaranth (4), hemp (5), quinoa (6), canihua (7), poppy (8), basil (9), black cumin (10), and psyllium (11). Additionally, well-defined bands in the 35–55 kDa range were observed in flaxseed, sesame, chia, amaranth, hemp, black cumin, and psyllium. By contrast, SDS-based extractions yielded a different banding pattern, characterized by a prominent band around 55 kDa in amaranth and intense bands between 15 and 35 kDa in flaxseed, sesame, chia, amaranth, hemp, quinoa, canihua, and poppy. TCA-based extraction resulted in a distinct profile as well, showing strong bands in the 35–55 kDa range for hemp, poppy, and black cumin, and bands between 15–35 kDa for most other species. No discernible bands were detected in basil seed extracts (Figure 3C). These findings further emphasize the significant impact of extraction methodology on protein solubilization and recovery.

To assess intra-species variability, the SDS extracts of all sample replicates for flaxseed, sesame, amaranth, hemp, quinoa, and poppy were also analyzed by SDS-PAGE. The corresponding gel images are provided in Figures S5–S9 (Supplementary Materials). Overall, the most intense bands, particularly those around 20 and 35–40 kDa, likely correspond to storage proteins such as globulins, which are composed of α- and β-subunits within these molecular weight ranges [36]. In flaxseed (Figure S5), prominent bands at ~20 and ~35 kDa probably represent the α- and β-chains of globulins, which constitute approximately 18.6% of total protein content, or alternatively albumins, which account for around 17.7% [9,36,50]. Sesame seed extracts (Figure S6) showed strong bands between 25 and 35 kDa and around 20 kDa, likely also corresponding to globulins, the predominant protein fraction (67.3%) [51]. Amaranth (Figure S7A) exhibited a similar banding profile, with additional strong bands around 70 kDa that may correspond to vicilin-like or embryonic DC-8-like proteins [52]. Hemp extracts (Figure S7B) showed pronounced bands at ~20 and ~35 kDa, characteristic of edestin, a hexameric 11S globulin [53]. Quinoa samples (Figure S8) displayed faint but consistent bands at ~20 and ~32 kDa, likely corresponding to 11S-type seed storage proteins. In poppy seed (Figure S9), the observed bands at ~20 and ~35 kDa may reflect globulins, while a band around 55 kDa may represent a phosphatase known to be specific to poppy seeds.

Overall, despite minor differences in band intensity, the SDS-PAGE profiles were highly consistent among samples of the same species, confirming the reproducibility and robustness of the standardized SDS extraction protocol across biological replicates.

### 2.4. Targeted Discovery and Validation of Species-Specific Peptide Markers

Building upon the optimized extraction protocols, this section details the systematic identification, selection, and preliminary validation of robust species-specific peptide markers, highlighting their potential for reliable superseed authentication using a targeted LC-MS/MS workflow.

#### 2.4.1. Identification of Robust Peptide Markers for Superseed Authentication

**Figure 4:**
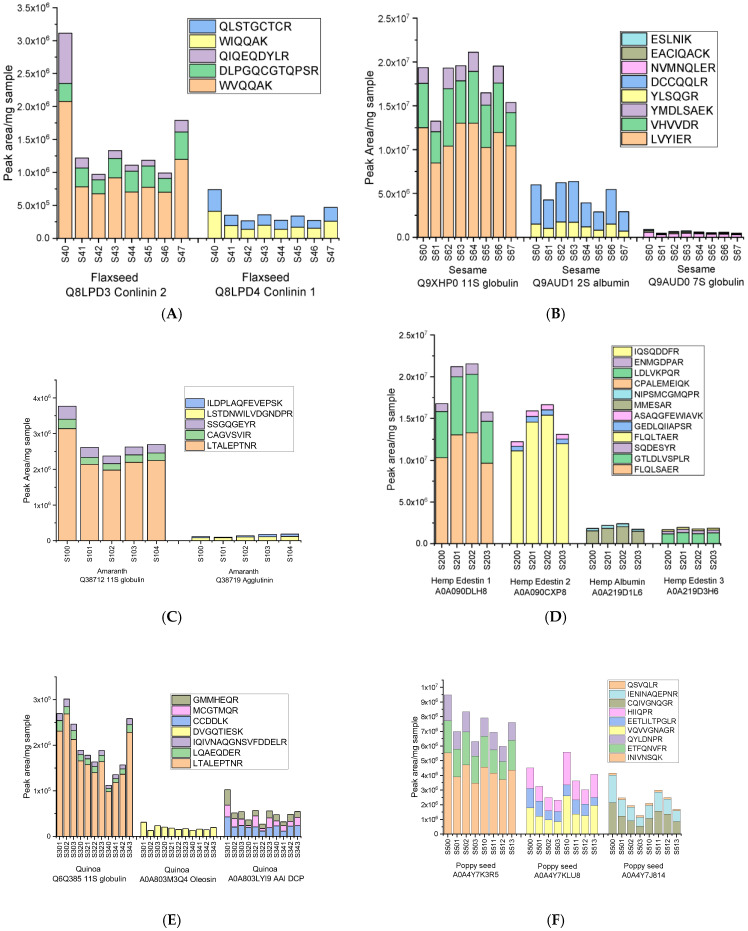
Distribution of potential biomarker peptides identified for (**A**) flaxseed storage proteins Q8LPD3 conlinin 2 and Q8LPD4 conlinin 1; (**B**) sesame storage proteins Q9XHP0 11S globulin, Q9AUD1 2S albumin, and Q9AUD0 7S globulin; (**C**) amaranth storage proteins Q38712 11S globulin and Q38719 agglutinin, (**D**) hemp proteins A0A090DLH8 edestin 1, A0A090CXP8 edestin 2, A0A219D1L6 albumin, and A0A219D3H6 edestin 3; (**E**) quinoa, including Q6Q385 11S seed storage globulin, A0A803M3Q4 oleosin, and A0A803LYI9, and (**F**) poppy seed, with proteins A0A4Y7J814, A0A4Y7K3R5, and A0A4Y7KLU8. The results are expressed as peak area values per mg of defatted seed flour.

In order to establish a robust approach for the differentiation and authentication of superseeds, a targeted mass-spectrometry strategy was implemented to identify species-specific peptide markers. These peptides were selected on the basis of (1) their uniqueness in complete seed proteomes, (2) both robustness (stability and minimal interference) and a strong signal (high abundance) for accurate and reliable detection in experimental samples, and (3) their association with taxonomically or functionally relevant proteins. Protein extracts from nine superseed species were analyzed: flax, sesame, amaranth, hemp, quinoa, poppy seeds, basil seed, cumin and psyllium seeds. All samples were processed according to the standardized protocol described above. The selected biomarker peptides for each successfully characterized seed are summarized in Table 1, including their parental proteins, peptide sequences and observed signal intensity ranges. In flaxseed, peptides derived from 2S albumins (conlinin 1 and 2) showed particularly strong signals. Among them, the tryptic peptide WVQQAK (derived from conlinin 2) was systematically detected with high intensity over several replicates (values ranging from 675,000 to 2,100,000 peak area/mg defatted flour) and was therefore selected as a potential main marker (Figure 4A). Other specific peptides such as DLPGQCGTQPSR and QIQEQDYLR were also identified and could be used as qualifiers for additional confirmatory assessments. In sesame extracts, eight specific target peptides from 11S, 2S and 7S globulins (Table 1) were successfully identified. The LVYIER peptide, derived from 11S globulin (Q9XHP0), showed the most robust and stable signal (10,170,000–12,958,000 peak area/mg defatted flour) and was selected as the main marker (Figure 4B). Complementary peptides were also successfully detected, including the 2S albumin peptide DCCQQLR (Q9AUD1) and the 11S globulin peptide VHVVDR (Q9XHP0), allowing further reinforcement of the species specificity.

In amaranth, the LTALEPTNR specific peptide derived from 11S globulin (Q38712) showed a high intensity in all the samples analyzed, ranging from 2,139,000 to 3,134,000 peak area/mg defatted flour (Figure 4C), while additional sequences including CAGVSVIR and SSGQGEYR from agglutinin-like lectin (Q38719) were also successfully detected, contributing further specificity. In hemp, several edestin isoforms (11S globulin-like proteins) produced strong signals, especially the peptides FLQLSAER and GTLDLVSPLR from edestin 1 (A0A090DLH8) and FLQLTAER from edestin 2 (A0A090CXP8) with signals ranging from 9,657,000 to 13,279,000; 4,987,000 to 7,015,000; and 11,128,000 to 15,372,000 peak area/mg defatted flour, respectively (Figure 4D). These peptides were therefore selected as potential co-markers due to their complementary sequence variation and consistent detection. Among the many proteins examined in quinoa, three were able to produce specific peptides that were successfully detected in all eleven samples analyzed (S301-S303, S320-S323, S340-S343). These included the peptide LTALEPTNR from 11S globulin (Q6Q385), which showed the highest intensity (98,000–269,000 peak area/mg defatted flour), as well as the peptides DVGQTIESK from oleosin (A0A803M3Q4) and CCDDLK from AAI protein (A0A803LYI9) (Figure 4E). In fact, although these last peptides showed more moderate intensities (in the range 12,000–43,000), they were still significant and were consequently selected as supporting peptides, contributing to the further distinction of quinoa. In the case of poppy seeds, where classical storage proteins did not yield specific peptides in the detectable range, it was still possible to identify and successfully measure specific peptides from other protein types in samples S500–S503 and S510–S513 (Figure 4F). The peptide INIVNSQK, specific to a cupin domain protein (A0A4Y7K3R5), for example, showed high intensity signals (ranging from 3,473,000 to 5,527,000 peak areas/mg defatted flour), thereby emerging as the most promising marker candidate for poppy seed authentication.

#### 2.4.2. Specificity and Stability of Selected Marker Peptides

In addition to their specificity, the potential marker peptides selected are also required to have good chemical stability for robust quantification, both in a wide variety of samples from different regions and cultivation conditions, as well as in complex mixed matrices or even processed products. For example, the peptide LVYIER derived from sesame 11S globulin not only provided excellent signal intensity, but also excellent stability compared with other peptides that, although specific, contained cysteine or tryptophan residues in their sequences that are more prone to oxidative modification. The observed variability in peptide marker intensities across samples (Table 1) likely reflects the inherent biological diversity among commercial seed samples, including differences in variety, growing conditions, maturity, and processing history. Additionally, matrix effects during LC-MS/MS analysis and the varying protein compositions between samples may contribute to this variability. While this variability is expected for authentic samples, it highlights the importance of establishing acceptance ranges for each marker during method validation. Another important parameter in the selection of potential biomarkers was the consideration of potential protein isoforms. In fact, isoform inferences tend to be noisy and may lead to the false detection of the targeted peptides [54,55]. It is well known that seed storage proteins may share similar amino acid sequences. For example, the isomeric peptides WVQQAK (conlinin 1) and WIQQAK (conlinin 2), and FLQLSAER (edestin 1) and FLQLTAER (edestin 2) were identified as potential markers in flaxseed and hemp, respectively. But as those isoforms are only present in the same species, there seems to be no inherent concern about using them to identify or authenticate the corresponding seeds. Interestingly, the recurrence of the peptide LTALEPTNR as a candidate marker in both amaranth and quinoa, two phylogenetically related pseudo-cereals, raises important concerns about its uniqueness when considering the combined proteome of all species investigated. This peptide originates from the tryptic digestion of homologous 11S globulin proteins in both seeds, yielding a precursor ion at *m*/*z* 507.7826++ and characteristic product ions at *m*/*z* 800.4261+, 729.3890+, 616.3049+, and 487.2623+. However, during cross-species analysis, the isomeric peptide LTALEPTNR was detected in both quinoa and amaranth, exhibiting overlapping retention times and indistinguishable MS/MS fragmentation patterns under the current analytical conditions. Due to this lack of specificity, it was ultimately excluded as a unique peptide marker. Furthermore, as part of the validation workflow, each candidate peptide was subjected to in silico cross-proteome analysis against available seed proteomes to assess potential homology. Additionally, we included blank extraction controls and cross-analyzed all seed species within the same batch runs to monitor for carryover and unintended detection in non-target species. No signals above background were detected for non-target seeds, supporting the specificity of the selected markers.

Overall, if seed storage proteins and especially 2S and 11S were found as sources of peptide markers for most seeds, poppy seeds represented a special case as no storage proteins here were found suitable to provide a specific peptide. Nevertheless, the identification of potential stable and reliable peptide markers from other protein groups, including proteins containing a cupin domain indicated the possibility of adapting the strategy by targeting less conventional proteins. This provides an interesting basis for extending the methodology to other seed species whose potential marker peptides were not successfully identified. In fact, chia, canihua, basil, black cumin and psyllium seeds were also investigated in the context of the present study but due to the lack of storage proteins fully annotated, the results obtained were not sufficient to allow the detection of reliable and reproducible peptide markers for these species. Another potential limitation could have been the levels or even the composition of the extracted proteins. For example, it can be seen from the electrophoresis gels (Figure 3) that samples S600 basil seed and S1000 psyllium seed exhibited very few or even no bands, irrespective of the extraction strategy. In this respect, further investigations on methodological optimizations in order to improve the protein extractability of the above-mentioned species need to be addressed, in order to achieve more in-depth proteomic coverage for their peptide markers’ identification. In this respect, an untargeted approach with independent data acquisition (DIA) could also be an interesting pathway together with the targeted approach, thus potentially broadening the spectrum of marker discovery for these species. The present study was designed as a proof-of-concept to establish the methodological framework using commercially available samples, which inherently limited sample diversity and numbers for some species. Future validation studies should incorporate larger sample sets with clearly defined biological replicates from diverse geographical origins and cultivation conditions to better characterize natural variability ranges and establish robust acceptance criteria for authentication purposes.

#### 2.4.3. Validation of the Targeted LC-MS/MS Method

To assess the robustness and reliability of the proposed targeted LC-MS/MS workflow, a validation study was performed on selected peptides representative of each seed species. The key validation parameters included internal standard (IS) recovery (Figure S10), repeatability (intra-day variation), reproducibility (inter-day variation), and linearity (Figures S11 and S12, Supplementary Materials). The summarized results are presented in Table 2. It can be observed that IS recovery values ranged from 89.0% to 97.6%, reflecting the low matrix effect across seed matrices. Repeatability values were consistently low (0.2–1.2% RSD), demonstrating high intra-assay precision, while reproducibility values did not exceed 7%, even in complex matrices like poppy and quinoa, confirming acceptable inter-assay performance. Linearity was also observed across all peptides investigated, with R^2^ values ranging from 0.9922 to 0.9998, which shows the quantitative reliability of the method. Overall, peptides such as WVQQAK (flaxseed), INIVNSQK (poppy), and YLSQGR (sesame) exhibited superior overall performance, with high IS recovery (>90%), low variability, and strong linearity, making them particularly suitable for authentication purposes. In contrast, slightly higher RSD values observed for peptides like IQIVNAQGNSVFDDLER (quinoa) may reflect matrix-related challenges such as signal suppression. These results collectively validate the analytical suitability of the selected peptide markers and support their implementation in further studies targeting food authentication, especially in real-world scenarios involving complex or processed seed products.

## 3. Materials and Methods

### 3.1. Biological Samples

In total, 81 commercially available superseeds were analyzed in this study, sourced from the Max Planck Institute of Molecular Plant Physiology (Potsdam, Germany). These different commercially available samples included eight flaxseeds (*Linum usitatissimum* L.), thirteen chia samples (*Salvia hispanica* L.), eight sesame samples (*Sesamum indicum* L.), five amaranth seeds (*Amaranthus* ssp.), four hemp samples (*Cannabis sativa* L.), eleven quinoa samples (*Chenopodium quinoa* Willd), four canihua samples (*Chenopodium pallidicaule* Aellen), eight poppy seeds (*Papaver somniferum* L.), four basil seeds (*Ocimum basilicum* L.), four cumin samples (*Nigella sativa* L.), and twelve psyllium seeds including four samples from *Plantago Afra* L. (black), four samples from *Plantago Indica* L. (black), and four samples from *Plantago ovata* F. (brown). Table S1 (see Supplementary Materials) provides an overview of the plant material information, and Figure S13 (Supplementary Materials) shows representative images of selected seeds, presented in both whole and ground forms. Visual comparison illustrates differences in seed morphology, color, and texture, which may influence protein extraction efficiency and analytical profiling.

### 3.2. Chemicals

Ammonium bicarbonate (AMBI), dithiothreitol (DTT), n-hexane, trichloroacetic acid (TCA), Tris(hydroxymethyl)aminomethane (Tris base), and Tris(2-carboxyethyl)phosphine hydrochloride (TCEP) were obtained from Carl Roth GmbH, Karlsruhe, Germany. Iodoacetamide (IAA) and urea were purchased from Sigma-Aldrich, Steinheim, Germany. Bovine serum albumin (BSA), used as a standard for protein quantification, was procured from Fluka Chemie AG, Buchs, Switzerland. Proteomics-grade trypsin for protein digestion was obtained from Amresco, Solon, OH, USA. Eluents used for mass spectrometry analysis were of LC-MS grade, and all other chemicals used in this study were of analytical grade, with a purity of 95% or higher.

### 3.3. Sample Preparation and Protein Extraction

All biological replicates of each seed type were first placed in Petri dishes and visually inspected for potential contamination. Due to the structural differences (shape and size) between the seeds investigated, several procedures were employed to obtain the seed flours. Milling of samples S40, S48, S60, S100, S200, S304, S400, S500, S600, and S900 (details are presented in Table S1) was performed at room temperature (RT) using an A10 analytical mill (IKA-Werke GmbH & CO. KG, Staufen, Germany). Amaranth samples (S101–S104) were ground with a Red Ruby coffee grinder using dry ice. All other samples were milled using a mortar and pestle under liquid nitrogen. The ground samples were stored at 4 °C throughout the testing period.

Before protein extraction, the samples were defatted by mixing 1 g of flour with 2 mL of n-hexane containing 0.05% butylhydroxytoluene. The mixture was shaken for 15 min at 60 rpm at room temperature and centrifuged at 10,000× *g* for 5 min at 4 °C. The supernatant was discarded, and this defatting step was repeated once. The samples were then air-dried under a fume hood. Fat content was calculated based on the difference in sample mass before and after defatting. The defatted samples were subsequently used for protein extraction.

Given the broad diversity of seed types and the known influence of buffer composition on protein solubility and extractability, three extraction methods were tested: (i) Ambi/urea buffer extraction, (ii) SDS buffer extraction, and (iii) TCA extraction. For this procedure, one sample from each of the seed types was randomly selected (not all samples could be used to optimize the sample preparation procedure) and used initially; and each extraction method was performed in triplicate. Subsequently, the method yielding the best protein recovery was selected as standard method for all samples.

#### 3.3.1. Protein Extraction Using the Ambi/Urea Method

Protein extraction using the Ambi/urea method involved mixing 100 mg of defatted sample material with 1 mL of extraction buffer (100 mM ammonium hydrogen carbonate, 4 M urea). The mixture was shaken for one hour at room temperature (80 rpm), followed by centrifugation at 10,000× *g* for 10 min at 4 °C. The resulting supernatant was carefully collected and transferred to fresh 1.5 mL reaction tubes. To further clarify the extract, the supernatant was incubated at −20 °C for 20 min and centrifuged again under the same conditions. The resulting clear supernatant containing the extracted proteins was transferred to new 1.5 mL tubes and stored at −20 °C until further use.

#### 3.3.2. SDS Buffer Extraction Method

For the SDS extraction method, 100 mg of defatted sample material were mixed with 1 mL of SDS extraction buffer (130 mM SDS, 50mM Tris-base, 130 mM DTT, pH 6.8) and incubated in a thermomixer at 50 °C with shaking at 1000 rpm for 1 h. Following incubation, the samples were cooled and centrifuged at 10,000× *g* for 5 min at 4 °C. The supernatant was transferred to fresh 2 mL reaction tubes, mixed with 1 mL of ice-cold acetone, and incubated at −20 °C for 20 min to precipitate proteins. After centrifugation, the supernatant was discarded, and the washing step was repeated using acetone. The protein pellet was air-dried and resuspended in 1 mL of Ambi/urea buffer. After an additional centrifugation step, the clear supernatants were collected and stored at −20 °C for subsequent analysis.

#### 3.3.3. TCA Extraction Method

The trichloroacetic acid (TCA) extraction method was performed following our previously described procedure with some modifications [56]. Briefly, 100 mg of defatted sample material was mixed with 1 mL of extraction buffer (50 mM Tris-HCl, 25 mM sucrose, 0.1 M EDTA, 1% Triton X-100, 10% glycerol, 10 mM DTT, pH 8) and incubated at room temperature for 1 h under gentle shaking (70 rpm). After centrifugation, the supernatants were mixed with cold 10% TCA in acetone and incubated at −20 °C for 1 h to precipitate the proteins. After a second centrifugation, the pellets were air-dried, resuspended in Ambi/urea buffer, and stored at −20 °C for further analysis.

### 3.4. Sample Characterization

#### 3.4.1. Protein Content

The crude protein content of some selected seed samples was determined using the Kjeldahl method, applying a nitrogen-to-protein conversion factor of 6.25 [57]. Due to practical limitations, total protein content was initially estimated on one representative sample per species. The concentration of extracted proteins in solution was measured using the Lowry method [58], with bovine serum albumin (BSA) used as a standard for calibration. Extraction yields, expressed as a percentage, were determined by taking into account the quantities of extracted proteins and the levels of total protein content obtained by the Kjeldahl method for each type of seed. This approach enabled a consistent comparison of extraction yields between different extraction methods and seed species.

#### 3.4.2. Electrophoretic Characterization

Protein profiling was performed using SDS-PAGE under reducing conditions. Separation was carried out on 10% bis-tris polyacrylamide gels prepared in-house [59,60]. For sample preparation, protein extracts were mixed in a 1:1 (*v*/*v*) ratio with 1× sample buffer containing 2% SDS, 5% β-mercaptoethanol, 10% glycerol, 0.01% bromophenol blue, and Tris buffer. The mixtures were heated at 100 °C for 5 min, cooled, and loaded onto gels alongside a protein marker using ~25 µg of protein per slot (PageRuler Plus Prestained Protein Marker, Thermo Fisher Scientific, Carlsbad, CA, USA). Electrophoresis was performed at 30 mA per gel for approximatively 90 min using a Criterion™ Vertical Electrophoresis Cell (Bio-Rad Laboratories, Singapore). After separation, gels were stained overnight in Coomassie Brilliant Blue R-250 solution followed by destaining in 10% acetic acid. The gels were then scanned (Bio-Professional VIS scanner, SERVA Electrophoresis GmbH, Heidelberg, Germany), and band profiles were analyzed using ImageLab software, version 6.01 (Bio-Rad Laboratories Ltd., Hertfordshire, UK).

### 3.5. Targeted Mass Spectrometry for Peptide Marker Search

#### 3.5.1. Sample Preparation

The samples were first subjected to trypsin digestion before mass spectrometry analysis. For this, the samples were diluted to 5 mg/mL protein and 400 µL were transferred into 1.5 mL reaction tubes. Reduction was achieved by adding 10 µL of 250 mM TCEP, followed by incubation at 50 °C for 20 min while shaking at 300 rpm. For alkylation, 10 µL of 250 mM IAA was added, vortexed, and incubated for an additional 20 min at 50 °C in the dark with shaking. Subsequently, 135 µL of digestion buffer (100 mM ammonium bicarbonate) and 20 µL of 4 mg/mL proteomics-grade trypsin were added resulting in a protein-to-enzyme ratio of 25:1 (*w*/*w*). The mixture was vortexed briefly and incubated overnight at 37 °C with shaking at 300 rpm. The digestion process was stopped by adding 15 µL of 40% formic acid (FA).

Following digestion, solid-phase extraction (SPE) was performed for sample cleanup. For this purpose, C18 columns were used, containing 300 mg of material each. The columns were activated with 6 mL of SPE buffer A (acetonitrile/water/formic acid in a 50:50:0.1, *v*/*v*/*v* ratio) and conditioned with 6 mL of 0.1% formic acid (FA). The digested proteins were then applied to the columns, washed with 6 mL of 0.1% FA, and finally eluted with 2 mL of SPE buffer B (acetonitrile/FA in a 100:0.1, *v*/*v* ratio), followed by 3 mL of 0.1% FA. The eluted samples were collected and transferred into glass vials for further analysis.

#### 3.5.2. Mass Spectrometric Analysis

A targeted mass spectrometric approach was employed to detect and relatively quantify potential peptide biomarkers specific to each superseed species. This strategy aimed to identify storage proteins and their corresponding tryptic peptides that could serve as analytical markers for species differentiation and potential quality control applications.

Multiple reaction monitoring (MRM) methods were developed for selected peptides based on their relevance, specificity, and detectability. The overall workflow followed the protocols previously described [61,62,63,64], adapted to the proteins of interest from each seed matrix.

The process started with a curated search in the online UniProt database to retrieve proteins representative of each superseed species. As only very few superseed proteins are reviewed or well annotated, unreviewed entries with annotation score ≥ 2/5 were considered. The amino acid sequences were imported into Skyline software, version 25.1.0.142 (MacCoss Lab, University of Washington, WA, USA) in FASTA format. In silico tryptic digestion was performed then with the following parameters: no missed cleavages; fixed carbamidomethylation; peptide length of 5–30 amino acids; and precursor charge state of +2. Transitions corresponding to singly charged b- and y-ions were selected for targeted monitoring. Whole proteomes of the respective species were used as background databases to assess peptide uniqueness via BLAST alignment (https://www.uniprot.org/blast, last access date: 7 July 2025), thus ensuring marker specificity.

Initially, 8–10 transitions with m/z values ranging from 200 to 1200 were defined per candidate peptide. After empirical evaluation, the four most intense and stable transitions per peptide were retained. The selection of MRM transitions was primarily based on signal intensity, signal-to-noise ratio, reproducibility across injections, and minimal interference in the background matrix. Collision energies were further optimized to enhance signal sensitivity. Peptide markers were prioritized based on four key criteria: (1) high and reproducible signal intensity, (2) good distribution among selected transitions, (3) chromatographic and spectral stability, and (4) minimal cysteine content to reduce variability due to oxidation. Two or three potential peptide markers per protein were then selected (one later used as quantifier and the other as qualifiers for confirmation).

The MRM method was applied using an Agilent (Santa Clara, CA, USA) Infinity 1260 LC system coupled to an Agilent G6470A Triple Quadrupole mass spectrometer operating in positive ionization mode. Peptide separation was performed on a Kinetex^®^ C8 reversed-phase column (Phenomenex, Torrance, CA, USA) at a flow rate of 0.5 mL/min, using 0.1% formic acid in water (solvent A) and 100% acetonitrile (solvent B). The gradient profile used for separation is summarized in Table S2 (see Supplementary Materials). Method validation included the assessment of linearity, accuracy, and reproducibility for each potential peptide marker using control samples. Skyline was used for raw data processing and peak area integration, and the relative quantification of protein content across the seed extracts is expressed as peak area per mg defatted flour. The final proteins selected are presented in Table S3 while the panel of the corresponding potential marker peptides, including sequences, their transitions, and retention times, is detailed in Tables S4–S8 (Supplementary Materials).

#### 3.5.3. Method Validation

To ensure the reliability and robustness of the targeted LC-MS/MS workflow, the method was validated for key performance parameters including internal standard (IS) recovery, repeatability, reproducibility, and linearity for representative peptide markers from each superseed species. Validation was performed using selected seed samples (e.g., S40 flaxseed, S100 amaranth, S200 hemp, S301 quinoa, S60 sesame, and S510 poppy seed).

IS recovery was determined by spiking a short peptide standard (GWGG) prior to the analysis and calculating the percentage recovery relative to the blanks.

Repeatability (intra-day precision) and reproducibility (inter-day precision) were assessed by analyzing multiple replicates (*n* = 3) across independent runs, expressed as relative standard deviation (RSD, %).

Linearity was evaluated by constructing calibration curves from serial dilutions of peptide standards and corresponding seed matrices, with coefficients of determination (R^2^) reported for each peptide.

Additionally, blank extraction controls were included in each batch to monitor for potential carryover, and cross-species analyses were systematically performed within the same runs to verify the absence of unintended detection in non-target species.

### 3.6. Statistical Analysis

All experiments were conducted in triplicate, and the results are expressed as the mean ± standard deviation. Statistical analysis was performed using GraphPad Prism 8 (GraphPad Software, Inc., San Diego, CA, USA), with significance assessed using two-way ANOVA followed by Tukey’s post hoc test. A *p*-value of <0.05 (two-sided) was considered as significant.

## 4. Conclusions

The aim of this study was to develop and implement a standardized workflow for the extraction, electrophoretic profiling and targeted mass spectrometric identification of specific peptide markers from a selection of nutrient-rich seeds, often referred to as “superseeds”. A comparative evaluation of different protein extraction protocols led to the development of an optimized method based on SDS buffer, enabling high-yield recovery of seed proteins for most of the seed species analyzed. SDS-PAGE profiling confirmed the reproducibility and efficiency of the extraction procedure. The identification of species-specific peptide markers was successfully achieved for 6 of the 11 superseeds investigated using a targeted HPLC-MS/MS strategy, with a focus on the abundant seed storage proteins, including peptides derived from conlinins in flaxseed, globulins in sesame, amaranth, quinoa and hemp, and cupin-like proteins in poppy seed. These results validate the potential of targeted proteomics to support the identification of unique peptide signatures for superseed authentication and traceability. However, the current strategy showed limitations when applied to chia, canihua, basil, black cumin and psyllium seeds under the current experimental conditions. These challenges underline the need for further research, in particular to improve proteomic coverage for these species. Before these peptide markers can be implemented for routine superseed authentication, comprehensive in-house method validation must be conducted to establish analytical performance parameters (precision, accuracy, detection limits) and demonstrate specificity against interferents and related species. Furthermore, validation in real-world applications including processed foods and commercial products is essential to confirm the practical utility of this approach. These steps will encompass spiking experiments in seed-derived products subjected to thermal or mechanical processing, the assessment of peptide stability under various processing conditions (e.g., roasting, extrusion), and the evaluation of potential matrix effects that may interfere with peptide detection. Additionally, complementary techniques, such as the incorporation of isotopically labeled internal standards, could further enhance the reliability of absolute quantification in complex food matrices. Ultimately, this targeted proteomic approach lays the foundations for a robust analytical approach supporting the authentication and traceability of superseeds and high-value products derived from superseeds.

## Figures and Tables

**Table 1:** Superseed species-specific peptide markers and their corresponding proteins identified using targeted LC-MS/MS.

Superseed Species	Protein Accession	Protein Name	Gene	Amino Acids	Mass (Da)	Marker Peptide	Intensity Range (Peak Area/mg)	Assigned Role
Flaxseed	Q8LPD3	Conlinin 2 (2S albumin)	Cnl2	168	19,012	WVQQAK	675,000–2,100,000	Quantifier
						DLPGQCGTQPSR	209,000–761,000	Qualifier
						QIQEQDYLR	82,000–271,000	Qualifier
	Q8LPD4	Conlinin 1 (2S albumin)	Cnl1	169	19,063	WIQQAK	137,000–410,000	Quantifier
						QLSTGCTCR	120,000–330,000	Qualifier
Sesame	Q9XHP0	11S globulin seed storage	n.d.	459	51,830	LVYIER	10,170,000–12,958,000	Quantifier
						VHVVDR	3,819,000–6,576,000	Qualifier
						YMDLSAEK	1,142,000–2,202,000	Qualifier
	Q9AUD1	2S albumin	LOC10 5174067	153	17,504	YLSQGR	620,000–1,655,000	Quantifier
						DCCQQLR	2,105,000–4,642,000	Qualifier
	Q9AUD0	7S globulin	n.d.	585	67,069	NVMNQLER	216,000–486,000	Quantifier
						EACIQACK	46,000–179,000	Qualifier
						ESLNIK	63,000–148,000	Qualifier
Amaranth	Q38712	11S globulin	n.d.	501	56,672	LTALEPTNR	2,139,000–3,134,000	Qualifier
						CAGVSVIR	179,000–268,000	Qualifier
						SSGQGEYR	216,000–362,000	Quantifier
	Q38719	Agglutinin	AHA	304	34,958	LSTDNWILVDGNDPR	80,000–107,000	Quantifier
						ILDPLAQFEVEPSK	18,000–69,000	Qualifier
						TYDGLVHIK	23,000–58,000	Qualifier
Hemp	A0A090DLH8	Edestin 1 (11S globulin)	ede1A	511	58,504	FLQLSAER	9,657,000–13,279,000	Quantifier
						GTLDLVSPLR	4,987,000–7,015,000	Qualifier
						SQDESYR	952,000–1,242,000	Qualifier
	A0A090CXP8	Edestin 2 (11S globulin)	ede2C	491	55,986	FLQLTAER	11,128,000–15,372,000	Quantifier
						GEDLQIIAPSR	535,000–676,000	Qualifier
						ASAQGFEWIAVK	548,000–662,000	Qualifier
	A0A219D1L6	Albumin (2S albumin)	Cs2S-1	142	16,742	MMESAR	1,487,000–2,039,000	Quantifier
						NIPSMCGMQPR	241,000–331,000	Qualifier
						CPALEMEIQK	17,000–31,000	Qualifier
	A0A219D3H6	Edestin 3 (11S globulin)	CsEde3B	491	55,938	LDLVKPQR	1,325,000–1,190,000	Quantifier
						ENMGDPAR	331,000–416,000	Qualifier
						IQSQDDFR	178,000–210,000	Qualifier
Quinoa	Q6Q385	11S seed storage globulin	11S	480	53,641	LTALEPTNR	98,000–269,000	Qualifier
						LQAEQDER	6000–23,000	Qualifier
						IQIVNAQGNSVFDDELR	7000–17,000	Quantifier
	A0A803M3Q4	Oleosin	n.d.	185	19,083	DVGQTIESK	13,000–31,000	Quantifier
	A0A803LYI9	AAI domain-containing protein	n.d.	129	15,358	CCDDLK	12,000–43,000	Quantifier
						MCGTMQR	3000–26,000	Qualifier
						GMMHEQR	8000–34,000	Qualifier
Poppy seed	A0A4Y7J814	Protein-serine/threonine phosphatase	C5167_ 014922	853	95,547	CQIVGNQGR	540,000–2,148,000	Qualifier
						IENINAQEPNR	602,000–1,870,000	Quantifier
						QSVQLR	96,000–141,000	Qualifier
	A0A4Y7K3R5	Cupin type-1 domain-containing protein	C5167_ 011661	468	53,356	INIVNSQK	3,473,000–5,527,000	Quantifier
						ETFQNVFR	1,232,000–2,253,000	Qualifier
						QYLDNPR	996,000–1,748,000	Qualifier
	A0A4Y7KLU8	Cupin type-1 domain-containing protein	C5167_ 048617	514	57,714	VQVVGNAGR	846,000–2,601,000	Quantifier
						EETLILTPGLR	546,000–1,332,000	Qualifier
						HIIQPR	777,000–2,231,000	Qualifier

For each seed species, the selected biomarker peptides are listed along with their parent protein accession numbers, protein names, and their assigned role as either primary marker or qualifier peptide. The range of observed signal intensities (expressed as peak area per mg defatted flour) is provided to indicate peptide detectability and relative abundance across biological replicates. n.d.: no data.

**Table 2:** Results of the method validation for some selected seed samples.

	Protein ID	Peptide	Validation Criterion			
			IS Recovery (%)	Repeatability (% RSD)	Reproducibility (% RSD)	Linearity (R^2^)
Amaranth	Q38712	SSGQGEYR	89.4 ± 1.4	0.2	1.1	0.9984
	Q38719	LSTDNWILVDGNDPR		2.5	5.9	0.9989
Flaxseed	Q8LPD3	WVQQAK	90.2 ± 1.7	0.1	3.3	0.9941
	Q8LPD4	WIQQAK		0.7	5.5	0.9982
Hemp	A0A090CXP8	FLQLTAER	97.6 ± 0.9	0.4	2.4	0.9986
	A0A090DLH8	FLQLSAER		0.7	4.8	0.999
	A0A219D1L6	MMESAR		0.2	0.8	0.9922
Poppy	A0A4Y7J814	IENINAQEPNR	93.7 ± 0.7	2.1	7.0	0.9993
	A0A4Y7K3R5	INIVNSQK		0.3	4.9	0.9985
	A0A4Y7KLU8	VQVVGNAGR		0.7	3.3	0.9967
Quinoa	A0A803LYI9	CCDDLK	90.0 ± 4.3	0.4	5.5	0.9985
	A0A803M3Q4	DVGQTIESK		0.8	8.2	0.9977
	Q6Q385	IQIVNAQGNSVFDDELR		5.8	7.5	0.9988
Sesame	Q9AUD1	YLSQGR	97.1 ± 1.1	8.5	6.4	0.9975
	Q9XHP0	LVYIER		1.2	1.2	0.9975

The results are presented for the peptides S40 flaxseed, S100 amaranth, S200 hemp, S301 quinoa, S60 sesame and S510 poppy seed. IS = internal standard. RSD = relative standard deviation.

## Data Availability

The original contributions presented in this study are included in the article/Supplementary Materials. Further inquiries can be directed to the corresponding author.

## Supplementary Materials

The following supporting information can be downloaded at: https://www.mdpi.com/article/10.3390/molecules30142993/s1, Figure S1: Fat content in representative samples of eleven selected superfoods; Figure S2: Distribution of fat content across all analyzed samples from (A) flaxseed, (B) sesame, (C) amaranth, (D) hemp, (E) quinoa, and (F) poppy seed, highlighting intra-species variability; Figure S3: Comparison between the protein content obtained from representative samples of eleven superfoods and corresponding values reported in the literature; Figure S4: Comparative analysis of protein concentration and extracted protein yield from the modified SDS extraction protocol, with and without the addition of TCEP and IAA, across flaxseed, sesame, and black cumin samples; Figure S5: SDS-PAGE of flaxseed samples from the final standardized SDS extraction method; Figure S6: SDS-PAGE of sesame samples from the final standardized SDS extraction method; Figure S7: SDS-PAGE of (A) amaranth samples and (B) hemp samples from the final standardized SDS extraction method; Figure S8: SDS-PAGE of all quinoa samples from the final standardized SDS extraction method; Figure S9: SDS-PAGE of all poppy seed samples from the final standardized SDS extraction method; Figure S10: Recovery of the internal standard GWGG in S40 flaxseed, S100 amaranth, S200 hemp, S301 quinoa, S60 sesame and S510 poppy seed compared to the blank; Figure S11: Linearity of quantifier peptides of (a) amaranth, (b) flaxseed and (c) hem seeds; Figure S12: Linearity of quantifier peptides of (a) poppy, (b) quinoa and (c) sesame seeds; Figure S13: Visual representation of whole and ground forms of selected seed samples used in this study; Table S1: List of the sample materials used in this study, including the different superfoods, their corresponding sample codes, color, origin, and the commercial producers; Table S2: Flow gradient of the final LC-MS/MS method; Table S3: Overview of the final set of proteins selected for targeted HPLC-MS/MS analysis for each superfood, aiming to identify potential biomarkers; Table S4: Parameters of the applied MRM method for identifying and quantifying potential biomarker peptides derived from tryptic digestion of selected sesame proteins; Table S5: Parameters of the applied MRM method for identifying and quantifying potential biomarker peptides derived from tryptic digestion of selected amaranth proteins; Table S6: Parameters of the applied MRM method for identifying and quantifying potential biomarker peptides derived from tryptic digestion of selected hemp proteins; Table S7: Parameters of the applied MRM method for identifying and quantifying potential biomarker peptides derived from tryptic digestion of selected quinoa proteins; Table S8: Parameters of the applied MRM method for identifying and quantifying potential biomarker peptides derived from tryptic digestion of selected poppy seed proteins.
