# Supplementary material for: Discovery of Species-Specific Peptide Markers for Superseed Authentication Using Targeted LC-MS/MS Proteomics
Source: Molecules. 2025 Jul 16;30(14):2993. doi: 10.3390/molecules30142993 (PMC12297901; doi:10.3390/molecules30142993)
Supplement: Supplementary file 1 [file molecules-30-02993-s001.zip › molecules-3738750-supplementary.pdf]

Supplementary Material

Discovery of Species-Specific Peptide Markers for Superseed Authentication Using Targeted LC-MS/MS Proteomics

Sorel Tchewonpi Sagu [1, *], Beatrice Schnepf [2], Peter Stenzel [3], Kapil Nichani [2], Alexander Erban [3], Joachim Kopka [3], Harshadrai M. Rawel [2], Andrea Henze [1]

1 Institute of Agricultural and Nutritional Sciences, Martin Luther University Halle-Wittenberg, Von-Danckelmann-Platz 2, 06120 Halle (Saale), Germany; andrea.henze@landw.uni-halle.de

2 Institute of Nutritional Science, University of Potsdam, Arthur-Scheunert-Allee 114-116, 14558 Nuthetal, Germany; bea.schnepf@gmail.com (B.S.); kapil.nichani@uni-potsdam.de (K.N.); hmrawel@yahoo.de (H.M.R.)

3 Max-Planck-Institut of Molecular Plant Physiology, Potsdam Science Park, Am Mühlenberg 1, 14476 Potsdam, Germany; p.stenzel@uke.de (P.S.); erban@mpimp-golm.mpg.de (A.E.); kopka@mpimp-golm.mpg.de (J.K.)

* Correspondence: sorel.sagu@landw.uni-halle.de; Tel.: +49-345-5522-650

Content

Figure S1. Fat content in representative samples of eleven selected superfoods.

Figure S2. Distribution of fat content across all analyzed samples from (A) flaxseed, (B) sesame, (C) amaranth, (D) hemp, (E) quinoa, and (F) poppy seed, highlighting intra-species variability.

Figure S3. Comparison between the protein content obtained from representative samples of eleven superfoods and corresponding values reported in the literature.

Figure S4. Comparative analysis of protein concentration and extracted protein yield from the modified SDS extraction protocol, with and without the addition of TCEP and IAA, across flaxseed, sesame, and black cumin samples.

Figure S5. SDS-PAGE of flaxseed samples from the final standardized SDS extraction method.

Figure S6. SDS-PAGE of sesame samples from the final standardized SDS extraction method.

Figure S7. SDS-PAGE of (A) amaranth samples and (B) hemp samples from the final standardized SDS extraction method.

Figure S8. SDS-PAGE of all quinoa samples from the final standardized SDS extraction method.

Figure S9. SDS-PAGE of all poppy seed samples from the final standardized SDS extraction method.

Figure S10. Recovery of the internal standard GWGG in the S40 flaxseed, S100 amaranth, S200 hemp, S301 quinoa, S60 sesame and S510 poppy seed compared to the in blank.

Figure S11. Linearity of quantifier peptides of (a) amaranth, (b) flaxseed and (c) hem seeds;

Figure S12. Linearity of quantifier peptides of (a) poppy, (b) quinoa and (c) sesame seeds;

Figure S13. Visual representation of whole (upper row) and ground forms (lower row) of selected seed samples used in this study.

Table S1. List of the sample materials used in this study, including the different superfoods, their corresponding sample codes, color, origin, and the commercial producers.

Table S2. Flow gradient of the final LC-MS/MS method

Table S3. Overview of the final set of proteins selected for targeted HPLC-MS/MS analysis for each superfood, aiming to identify potential biomarkers

Table S4. Parameters of the applied MRM method for identifying and quantifying potential biomarker peptides derived from tryptic digestion of selected sesame proteins

Table S5. Parameters of the applied MRM method for identifying and quantifying potential biomarker peptides derived from tryptic digestion of selected amaranth proteins

Table S6. Parameters of the applied MRM method for identifying and quantifying potential biomarker peptides derived from tryptic digestion of selected hemp proteins

Table S7. Parameters of the applied MRM method for identifying and quantifying potential biomarker peptides derived from tryptic digestion of selected quinoa proteins

Table S8. Parameters of the applied MRM method for identifying and quantifying potential biomarker peptides derived from tryptic digestion of selected poppy seed proteins

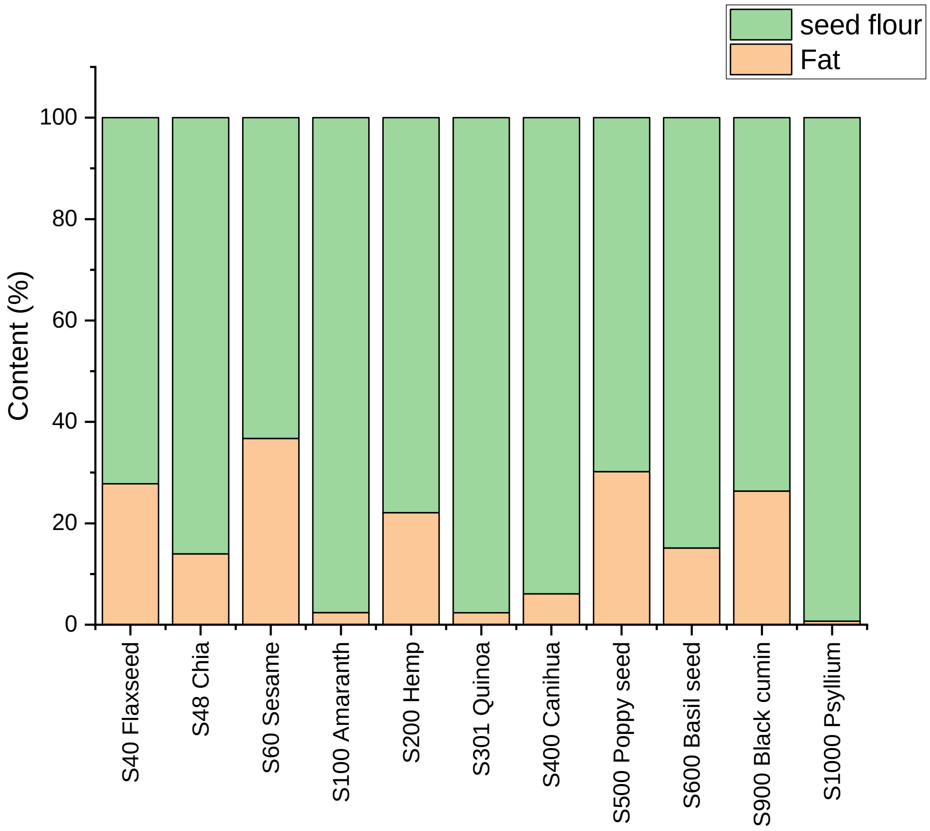

Figure S1. Fat content in representative samples of eleven selected superfoods.

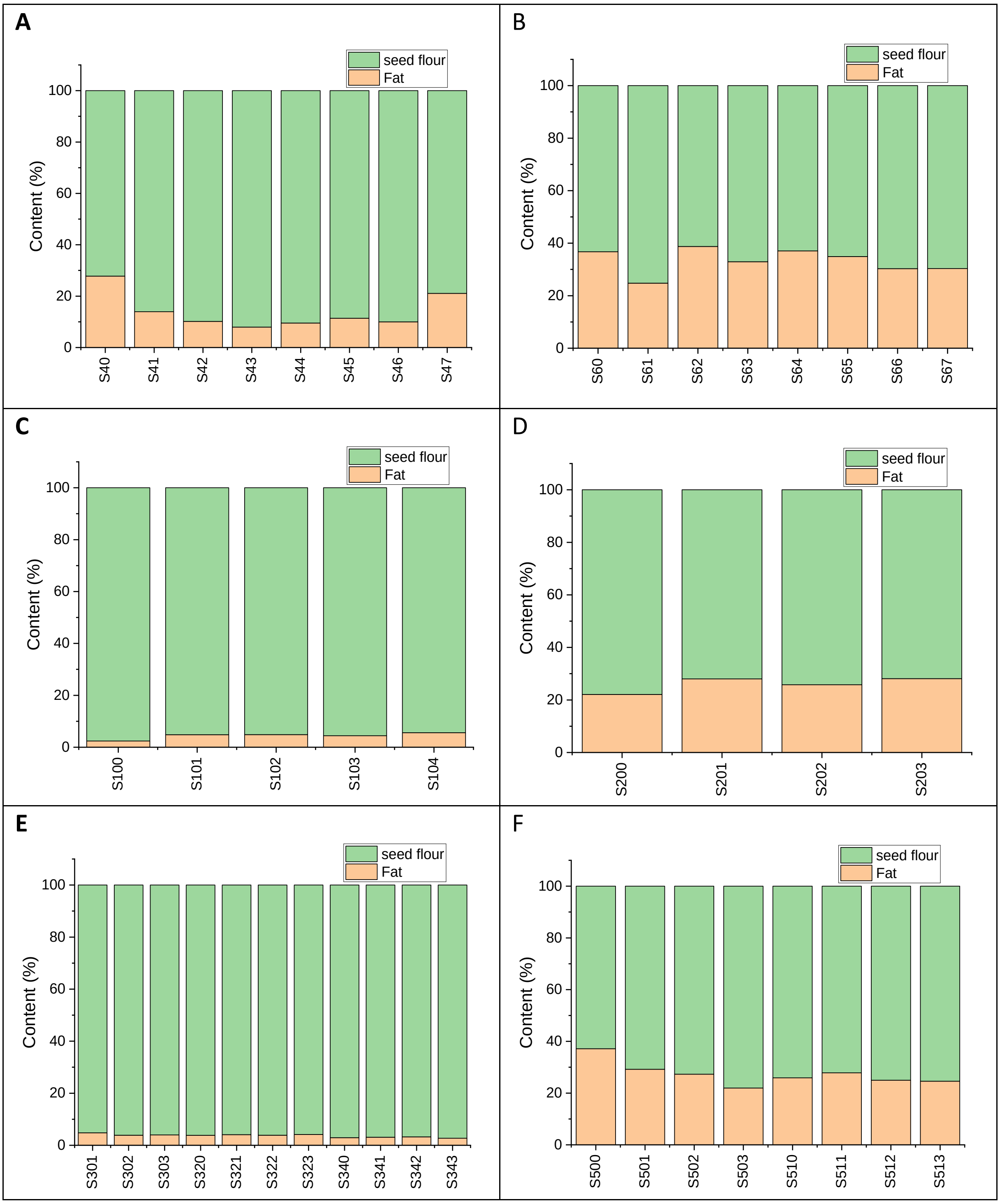

Figure S2. Distribution of fat content across all analyzed samples from (A) flaxseed, (B) sesame, (C) amaranth, (D) hemp, (E) quinoa, and (F) poppy seed, highlighting intra-species variability.

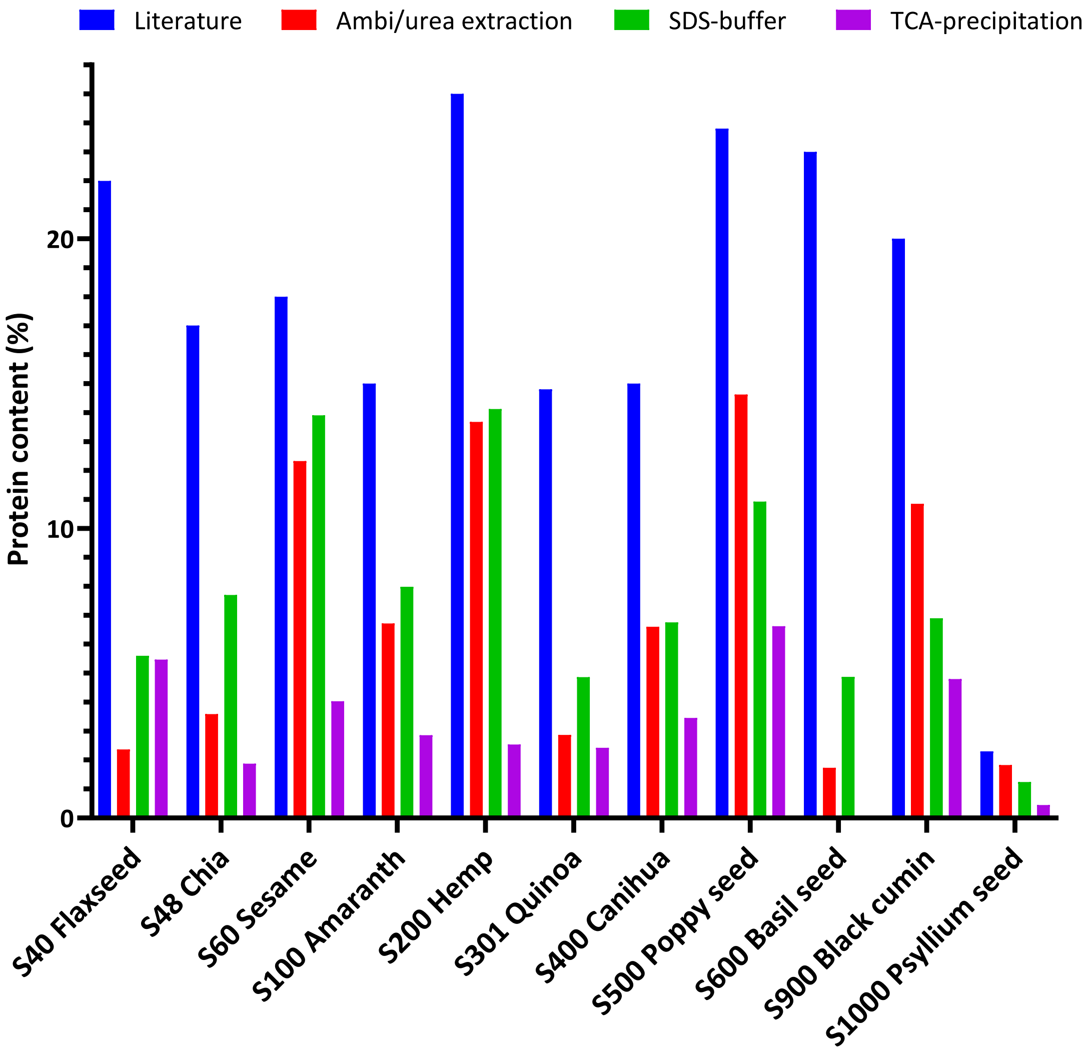

Figure S3. Comparison between the protein content obtained from representative samples of eleven superfoods and corresponding values reported in the literature.

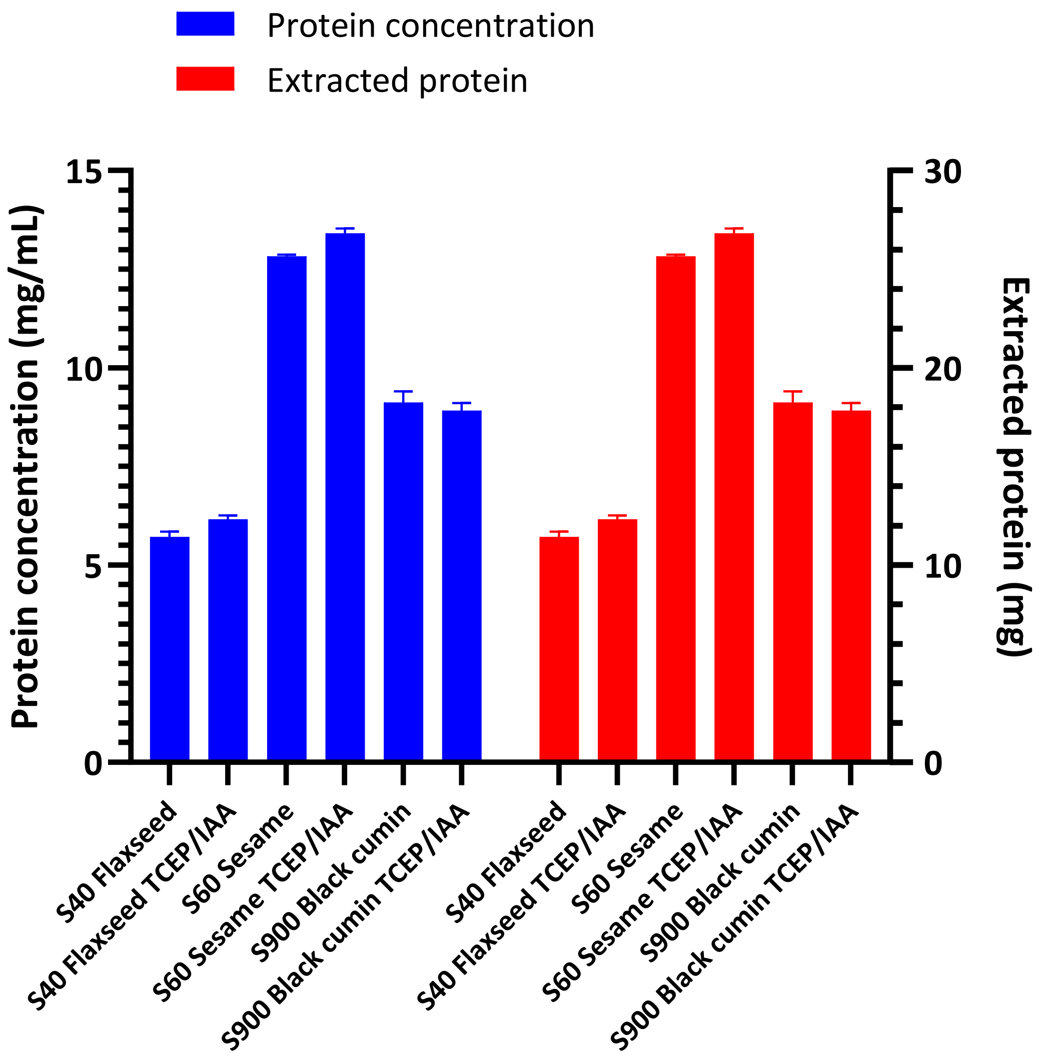

Figure S4. Comparative analysis of protein concentration and extracted protein yield from the modified SDS extraction protocol, with and without the addition of TCEP and IAA, across flaxseed, sesame, and black cumin samples.

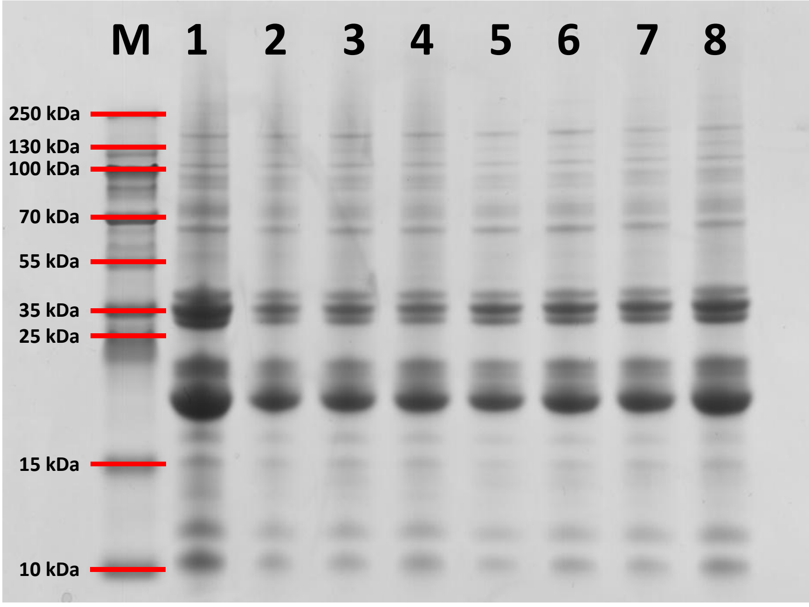

Figure S5. SDS-PAGE of flaxseed samples (S40–S47) from the final standardized SDS extraction method. (M) Marker, 1- S40, 2- S41, 3- S42, 4- S43, 5- S44, 6- S45, 7- S46, 8- S47 flaxseed

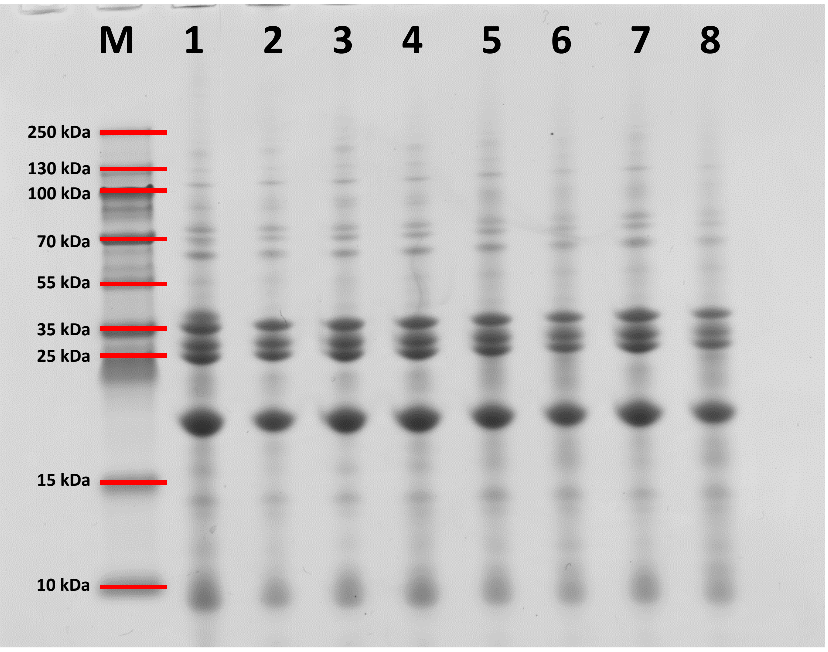

Figure S6. SDS-PAGE of sesame samples (S60–S67) from the final standardized SDS extraction method. (M) Marker, 1- S60, 2- S61, 3- S62, 4- S63, 5- S64, 6- S65, 7- S66, 8- S67 sesame.

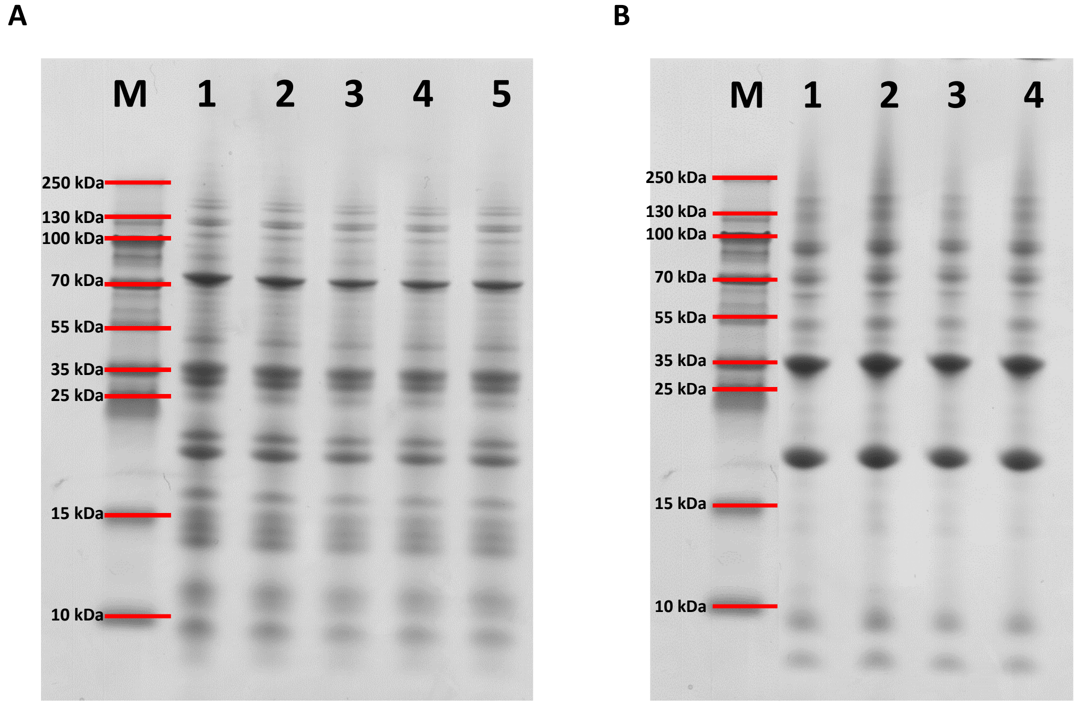

Figure S7. SDS-PAGE of (A) amaranth samples (S100–S104) and (B) hemp samples (S200–S203) from the final standardized SDS extraction method. A: M- Marker, 1- S100 amaranth, 2- S101 amaranth, 3- S102 amaranth, 4- S103 amaranth, 5- S104 amaranth. B: M- Marker, 1- S200 hemp (1), 2- S201 hemp (2), 3- S202 hemp (3), 4- S203 hemp (4).

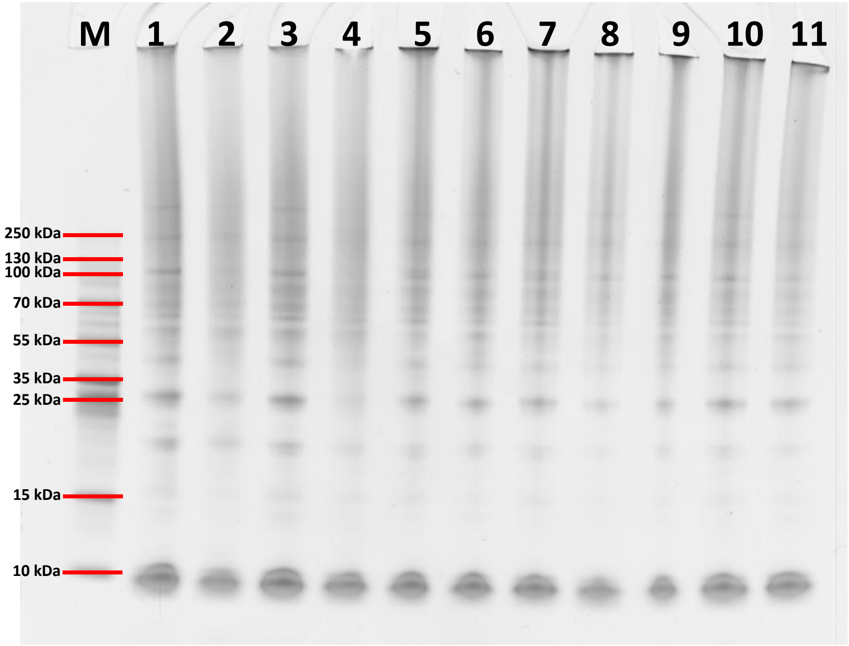

Figure S8. SDS-PAGE of all quinoa samples (S301–S303, S320–S323, S340–S343) from the final standardized SDS extraction method. M- Marker, 1- S301 Quinoa, 2- S302 Quinoa, 3- S303 Quinoa, 4- S320 Quinoa, 5- S321 Quinoa, 6- S322 Quinoa, 7- S323 Quinoa, 8- S340 Quinoa, 9- S341 Quinoa, 10- S342 Quinoa, 11- S343 Quinoa.

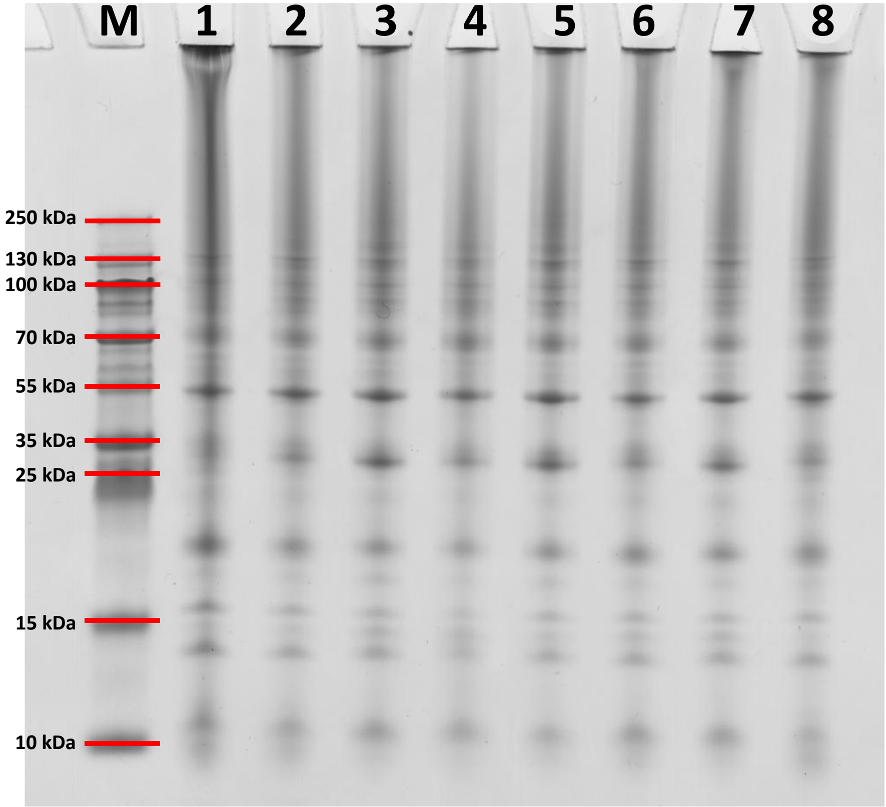

Figure S9. SDS-PAGE of all poppy seed samples (S500–S503, S510–S513) from the final standardized SDS extraction method. M- Marker, 1- S500 poppy, 2- S501 poppy, 3- S502 poppy, 4- S503 poppy, 5- S510 poppy, 6- S511 poppy, 7- S512 poppy, 8- S513 poppy.

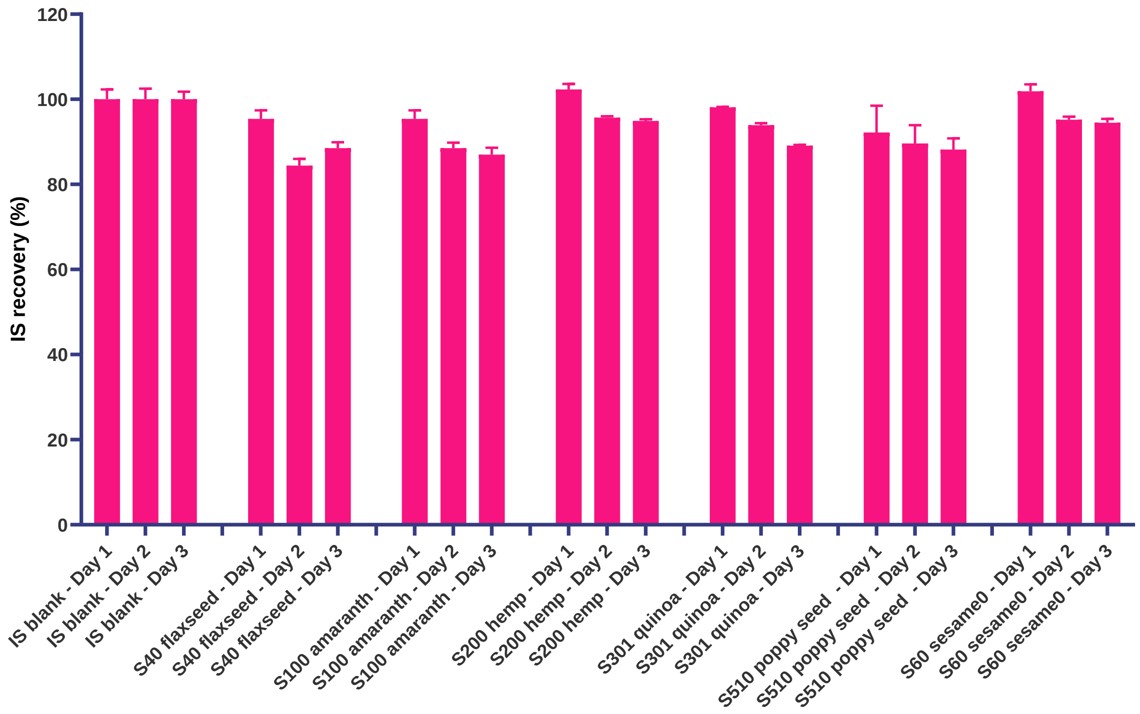

Figure S10. Recovery of the internal standard GWGG in the S40 flaxseed, S100 amaranth, S200 hemp, S301 quinoa, S60 sesame and S510 poppy seed compared to the in blank. Experiments were performed in three different days and each in triplicate.

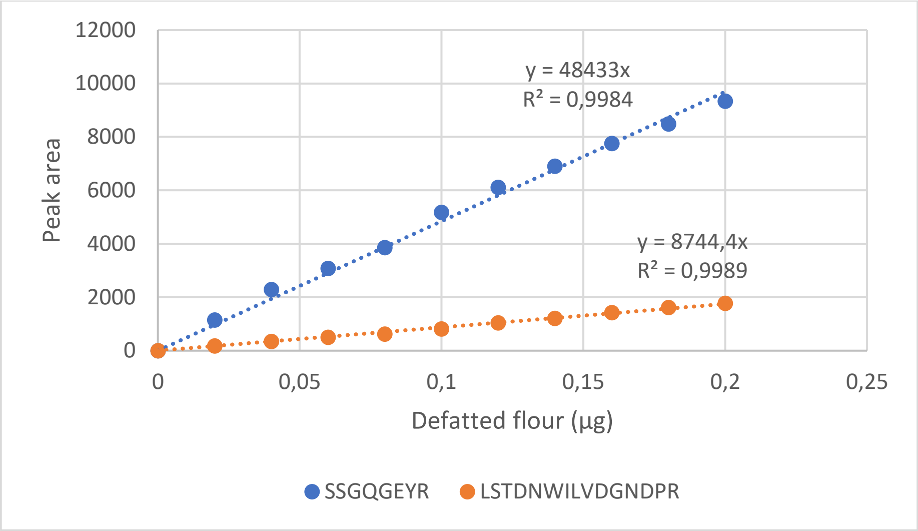

(a)

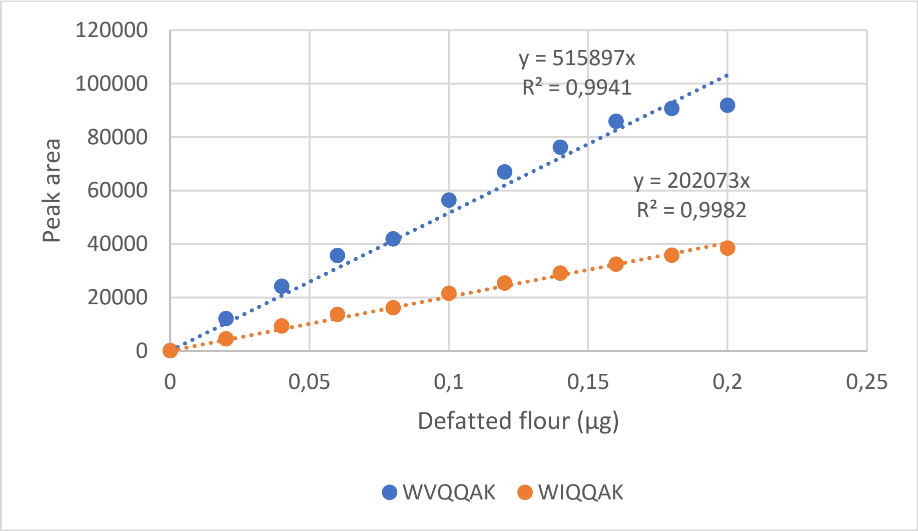

(b)

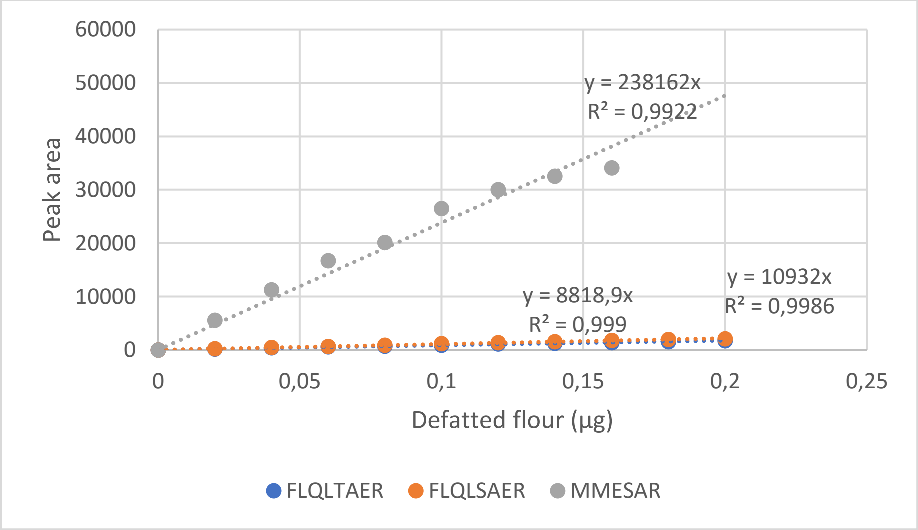

(c)

Figure S11. Linearity of quantifier peptides of (a) amaranth, (b) flaxseed and (c) hemp seeds

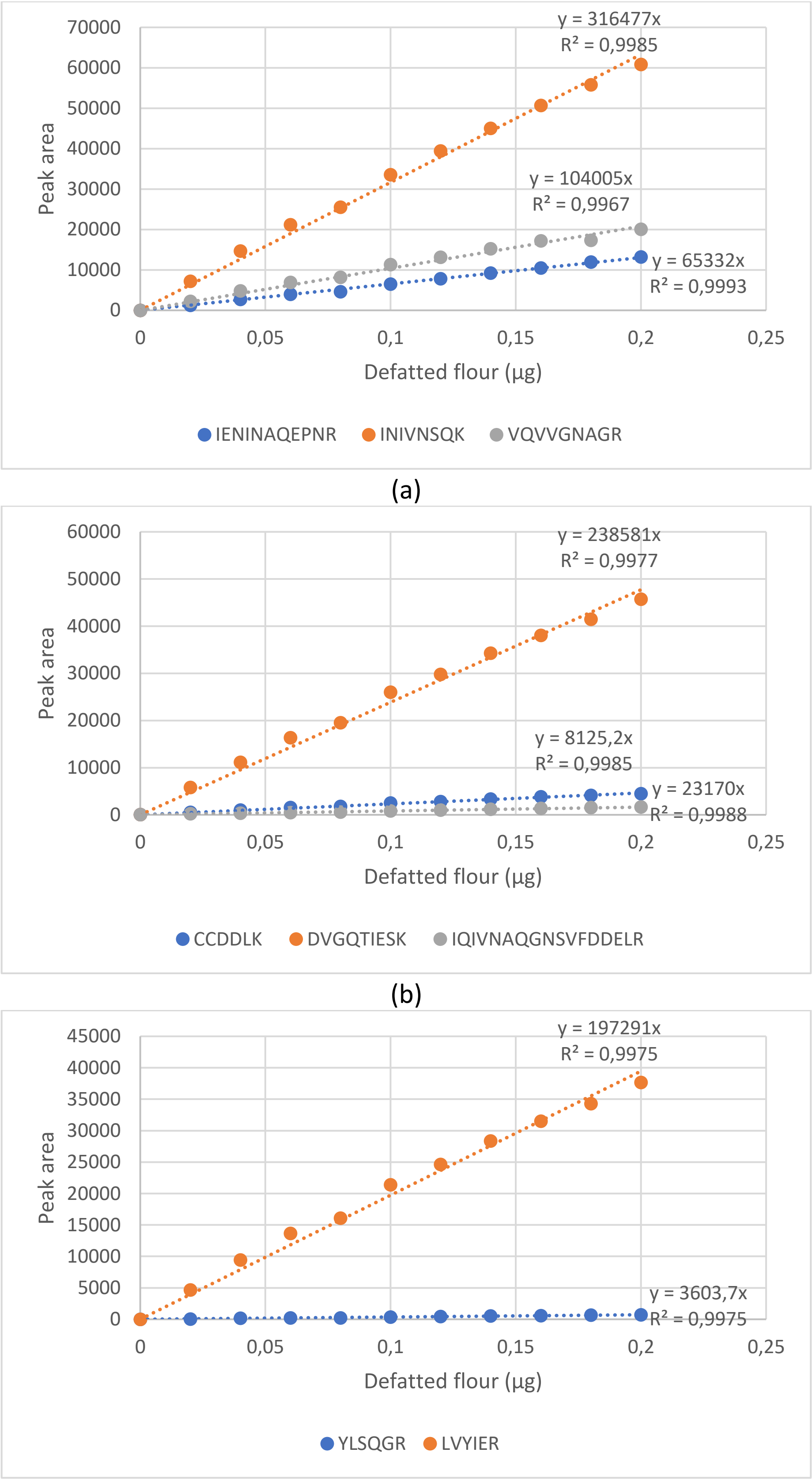

(c)

Figure S12. Linearity of quantifier peptides of (a) poppy, (b) quinoa and (c) sesame seeds

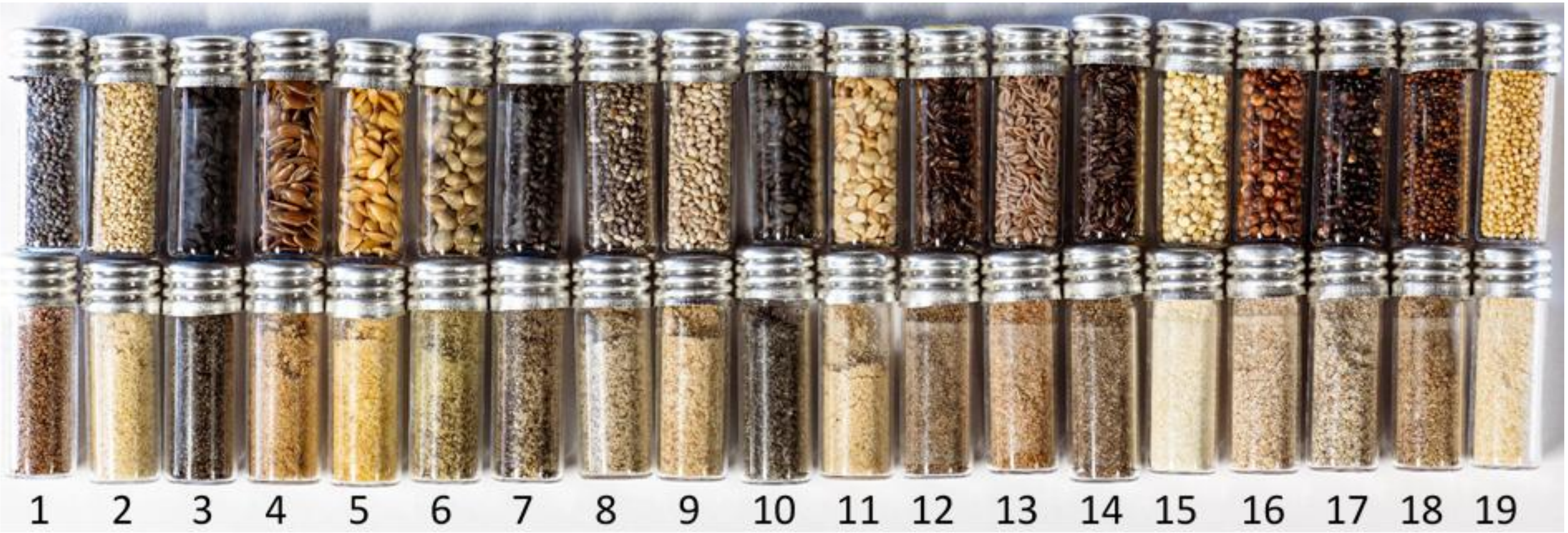

Figure S13. Visual representation of whole and ground forms of selected seed samples used in this study. Seed types include: (1) blue-gray poppy, (2) white poppy, (3) black cumin, (4) brown flaxseed, (5) golden yellow flaxseed, (6) gray-brown hemp, (7) black basil, (8) dark brown chia, (9) white chia, (10) black sesame, (11) white sesame, (12) black psyllium, (13) brown psyllium, (14) black psyllium, (15) white quinoa, (16) red quinoa, (17) black quinoa, (18) red canihua, and (19) white amaranth.

Table S1. List of the sample materials used in this study, including the different superfoods, their corresponding sample codes, color, origin, and the commercial producers.

Species	Ref. Number	Seed color	Product company	Origin
Flaxseed	S40	brown	Rapunzel Naturkost	Italy
	S41	brown	Antersdorfer Mühle GmbH & Vertriebs KG	Poland
	S42	brown	Davert GmbH	Czech Republic
	S43	brown	Spielberger GmbH	Origin unk
	S44	golden yellow	Rapunzel Naturkost	Kazakhstan
	S45	golden yellow	Spielberger GmbH	Hungary
	S46	golden yellow	dm drogerie markt GmbH	China
	S47	golden yellow	Carl Wilhem Clasen GmbH	China
Chia	S48	dark brown	Govinda Natur GmbH	Origin unk
	S49	dark brown	Davert GmbH	Nicaragua
	S50	dark brown	Davert GmbH	Uganda
	S51	dark brown	Rapunzel Naturkost	Paraguay
	S52	dark brown	dm drogerie markt GmbH	Paraguay
	S53	dark brown	Märsch Importhandels GmbH	Origin unk
	S54	dark brown	Herbert Kluth GmbH & Co. KG	Origin unk
	S55	dark brown	Midsona Germany GmbH	Origin unk
	S56	cream	Govinda Natur GmbH	Origin unk
	S57	cream	Kornwestheimer Bio-Marketing GmbH	Origin unk
	S58	cream	Tilia/Marulo	Argentinian
	S59	cream	Davert GmbH	Origin unk
	S68	dark brown	Duria Global GmbH & Co. KG	Paraguay
Sesame	S60	white	Bohlsener Mühle GmbH & Co. KG	Uganda
	S61	white	Davert GmbH	Pakistan
	S62	white	Rapunzel Naturkost	Egypt
	S63	white	Spielberger GmbH	Egypt
	S64	black	Govinda Natur GmbH	Origin unk
	S65	black	Arche Naturprodukte GmbH	Origin unk
	S66	black	ReformKontor GmbH & Co. KG	India
	S67	black	TerraSana	Belarus
Amaranth	S100	white	Bohlsener Mühle GmbH & Co. KG	India
	S101	white	Dennree GmbH	India
	S102	white	Rapunzel Naturkost	India
	S103	white	Carl Wilhem Clasen GmbH	India
	S104	white	Rila Feinkost-Importe GmbH und Co. KG	Origin unk
Hemp	S200	brown gray	Laia´s Predeinhanf GmbH	Origin unk
	S201	brown gray	Davert GmbH	Germany
	S202	brown gray	Chiron W. Misslisch/L.Stury GbR	Origin unk
	S203	brown gray	ReformKontor GmbH & Co. KG	China

Table S1. *Cont.*

Species	Ref. Number	Seed color	Product company	Origin
Quinoa	S301	white	Antersdorfer Mühle GmbH & Vertriebs KG	Origin unk
	S302	white	Bohlsener Mühle GmbH & Co. KG	Germany
	S303	white	Davert GmbH	Origin unk
	S320	red	Rapunzel Naturkost	Origin unk
	S321	red	Davert GmbH	Origin unk
	S322	red	EKIBIO	Bolivia
	S323	red	Rila Feinkost-Importe GmbH und Co. KG	Origin unk
	S340	black	Davert GmbH	Origin unk
	S341	black	EKIBIO	Bolivia
	S342	black	Rila Feinkost-Importe GmbH und Co. KG	Origin unk
	S343	black	Reishunger GmbH	Peru
Canihua	S400	red	Carl Wilhem Clasen GmbH	Bolivia
	S401	red	Schnitzer GmbH & Co. KG	Origin unk
	S402	red	Davert GmbH	Origin unk
	S403	red	Reishunger GmbH	Peru
Poppy seeds	S500	Blue-gray	Rapunzel Naturkost	Origin unk
	S501	Blue-gray	Davert GmbH	Turkey
	S502	Blue-gray	Seeberger GmbH	Origin unk
	S503	Blue-gray	Albert Ménéz	Origin unk
	S510	white	Sonnentor GmbH	Austria
	S511	white	TRS´Wholesale Co. Ltd.	Turkey
	S512	white	Jalpur MIllers	India
	S513	white	Mohnhof Gressl	Austria
Basil seeds	S600	black	TRS´Wholesale Co. Ltd.	India
	S602	black	Jalpur Millers	India
	S603	black	Gesund & Leben Elke Weintraut	Uzbekistan
	S604	black	Gesund & Leben Elke Weintraut	Vietnam
Black cumin	S900	black	TRS´Wholesale Co. Ltd.	India
	S901	black	Wagner Gewürze GmbH	Egypt
	S902	black	Gewürzmühle Brecht GmbH	Egypt
	S903	black	Bremer Gewürzhandel GmbH	Egypt
Psyllium seeds	S1000	black	Sonnentor GmbH	Austria
	S1001	black	Tee und Spezialitätenversand	Origin unk
	S1002	black	Aleavedis Naturprodukte GmbH	Origin unk
	S1003	black	Golden Peanut	Origin unk
	S1010	brown	TopFruits	Origin unk
	S1011	brown	Tee und Spezialitätenversand	Origin unk
	S1012	brown	Aurica	India
	S1013	brown	Naturprodukte Lembcke GbR	India
	S1020	black	TopFruits	Origin unk
	S1021	black	Herbis Natura	Austria
	S1022	black	YouHerbit/Konstantinos Spiliadis	Origin unk
	S1023	black	Edel Kraut GmbH	Origin unk

Table S2. Flow gradient of the final LC-MS/MS method

Time (min)	Eluent A (%)	Eluent B (%)
0	100	0
2	100	0
18	50	50
18.1	5	95
22	5	95
22.1	100	0
28	100	0

Table S3. Overview of the final set of proteins selected for targeted HPLC-MS/MS analysis for each superfood, aiming to identify potential biomarkers

	UniProt entry number	Name	Organisms	Gen	Length (AA)	Mass (Da)	Status
**Flaxseed**	Q8LPD3	Conlinin 2	Linum usitatissimum (Flax)	cnl2	168	19,012	unreviewed
	Q8LPD4	Conlinin 1	Linum usitatissimum (Flax)	cnl1	169	19,063	unreviewed
**Sesame**	Q9XHP0	11S globulin seed storage protein 2	Sesamum indicum (Oriental sesame)	n.a.	459	51,830	Reviewed
	Q9AUD1	2S albumin	Sesamum indicum (Oriental sesame)	LOC105174067	153	17,504	unreviewed
	Q9AUD0	7S globulin	Sesamum indicum (Oriental sesame)	n.a.	585	67,069	unreviewed
**Amaranth**	Q38712	11S globulin seed storage protein	Amaranthus hypochondriacus (Prince-of-Wales feather)	n.a.	501	56,672	unreviewed
	Q38719	Agglutinin	Amaranthus hypochondriacus (Prince-of-Wales feather)	AHA	304	34,958	unreviewed
**Hemp**	A0A090DLH8	Edestin 1	Cannabis sativa (Hemp) (Marijuana)	ede1A	511	58,504	unreviewed
	A0A090CXP8	Edestin 2	Cannabis sativa (Hemp) (Marijuana)	ede2C	491	55,986	unreviewed
	A0A219D1L6	Albumin	Cannabis sativa (Hemp) (Marijuana)	Cs2S-1	142	16,742	unreviewed
	A0A219D3H6	Edestin 3	Cannabis sativa (Hemp) (Marijuana)	CsEde3B	491	55,938	unreviewed
**Quinoa**	Q6Q385	11S seed storage	Chenopodium quinoa (Quinoa)	11S	480	53,641	unreviewed
	A0A803M3Q4	Oleosin	Chenopodium quinoa (Quinoa)	n.a.	185	19,083	unreviewed
	A0A803LYI9	AAI domain-containing protein	Chenopodium quinoa (Quinoa)	n.a.	129	15,358	unreviewed
**Poppy seed**	A0A4Y7J814	Protein-serine/threonine phosphatase	Papaver somniferum (Opium poppy)	C5167_ 014922	853	95,547	unreviewed
	A0A4Y7K3R5	Cupin type-1 domain-containing protein	Papaver somniferum (Opium poppy)	C5167_ 011661	468	53,356	unreviewed
	A0A4Y7KLU8	Cupin type-1 domain-containing protein	Papaver somniferum (Opium poppy)	C5167_ 048617	514	57,714	unreviewed

n.a: not available

Table S4. Parameters of the applied MRM method for identifying and quantifying potential biomarker peptides derived from tryptic digestion of selected sesame proteins

Entry number	Peptide sequence	Q1-Mass	Q3-Mass	Retention time (min)
Q9XHP0	R.LVYIER.G	396.7343	679.3774<br>580.3089<br>417.2456<br>213.1598	10.0
	R.VHVVDR.N	362.7087	488.2827<br>389.2143<br>237.1346<br>336.2030	6.7
	K.YMDLSAEK.G	478.7233	793.3760<br>662.3355<br>547.3086<br>295.1111	9.7
Q9AUD1	R.YLSQGR.S	362.1928	560.3151<br>447.2310<br>232.1404<br>277.1547	7.2
	R.DCCQQLR.N	490.2078	704.3508<br>544.3202<br>288.2030<br>276.0649	7.4
Q9AUD0	R.NVMNQLER.E	502.2531	M790.3876<br>659.3471<br>545.3042<br>345.1591	10.0
	K.EACIQACK.E	490.2204	619.3232<br>506.2391<br>378.1806<br>307.1435	7.4
	R.ESLNIK.Q	352.2029	487.3239<br>374.2398<br>260.1969<br>217.0819	9.0

Table S5. Parameters of the applied MRM method for identifying and quantifying potential biomarker peptides derived from tryptic digestion of selected amaranth proteins

Entry number	Peptide sequence	Q1-Mass	Q3-Mass	Retention time (min)
Q38712	R.LTALEPTNR.I	507.7826	800.4261<br>729.3890<br>616.3049<br>487.2623	10.8
	R.CAGVSVIR.R	431.2342	630.3933<br>573.3719<br>474.3035<br>387.2714	10.8
	R.SSGQGEYR.R	442.1989	796.3584<br>709.3264<br>652.3049<br>709.2788	9.1
Q38719	R.LSTDNWILVDGNDPR.E	857.9234	998.5265<br>885.4425<br>772.3584<br>673.2900	13.8
	K.ILDPLAQFEVEPSK.T	793.4272	963.4782<br>460.2402<br>331.1976<br>342.2023	14.9
	K.TYDGLVHIK.S	523.2875	781.4567<br>666.4297<br>496.3242<br>397.2558	11.4

Table S6. Parameters of the applied MRM method for identifying and quantifying potential biomarker peptides derived from tryptic digestion of selected hemp proteins

Entry number	Peptide sequence	Q1-Mass	Q3-Mass	Retention time (min)
A0A090DLH8	R.FLQLSAER.G	482.27	816.4574<br>703.3733<br>575.3148<br>462.2307	11
	K.GTLDLVSPLR.S	535.8139	799.4672<br>684.4403<br>571.3562<br>385.2558	12.6
	R.SQDESYR.Q	442.69	669.2838<br>554.2569<br>425.2143<br>460.1674	6.6
A0A090CXP8	R.FLQLTAER.G	489.27	717.3890<br>589.3304<br>375.1987	11.3
	R.GEDLQIIAPSR.I	599.8250	784.4676<br>656.4090<br>359.2037<br>656.3250	11
	K.ASAQGFEWIAVK.T	653.84	949.5142<br>745.4243<br>990.4680	12.9
A0A219D1L6	R.MMESAR.N	362.66	593.2712<br>333.1881<br>392.1308	7
	R.CPALEMEIQK.E	609.7965	961.5023<br>890.4652<br>777.3811<br>648.3385	10.9
	R.NIPSMCGMQPR.T	645.79	966.3954<br>879.3634<br>531.2708	10.4
A0A219D3H6	K.LDLVKPQR.S	484.7980	740.4777<br>627.3937<br>400.2303	9.1
	K.IQSQDDFR.G	504.74	895.3904<br>767.3319<br>680.2998	8.5
	R.ENMGDPAR.A	445.20	646.2977<br>515.2572<br>458.2358<br>343.2088	7.4

Table S7. Parameters of the applied MRM method for identifying and quantifying potential biomarker peptides derived from tryptic digestion of selected quinoa proteins

Entry number	Peptide sequence	Q1-Mass	Q3-Mass	Retention time (min)
Q6Q385	R.LTALEPTNR.I	507.7826	800.4261<br>729.3890<br>616.3049<br>487.2623	10.7
	K.LQAEQDER.G	494.7383	747.3268<br>676.2897<br>547.2471<br>304.1615	8.4
	R.IQIVNAQGNSVFDDELR.Q	959.4869	1151.5327<br>980.4684<br>794.3679<br>647.2995	13.8
A0A803M3Q4	K.DVGQTIESK.A	488.7509	762.3992<br>705.3777<br>577.3192<br>363.1874	9.7
A0A803LYI9	K.CCDDLK.M	405.6597	650.2814<br>260.1969<br>321.0686<br>551.1225	8.6
	R.MCGTMQR.K	442.1828	752.3178<br>592.2872<br>535.2657<br>434.2180	8.8
	K.GMMHEQR.M	444.6944	700.3195<br>569.2790<br>432.2201<br>303.1775	8.6

Table S8. Parameters of the applied MRM method for identifying and quantifying potential biomarker peptides derived from tryptic digestion of selected poppy seed proteins

Entry number	Peptide sequence	Q1-Mass	Q3-Mass	Retention time (min)
A0A4Y7J814	R.CQIVGNQGR.Q	516.2562	743.4159<br>630.3318<br>531.2634<br>402.1806	9.1
	R.IENINAQEPNR.R	649.3284	1055.5228<br>828.3959<br>643.3158<br>386.2146	9.8
	R.QSVQLR.G	365.7139	602.3620<br>515.3300<br>416.2616<br>288.2030	9.5
A0A4Y7K3R5	R.INIVNSQK.L	458.2665	802.4417<br>688.3988<br>575.3148<br>476.2463	10.1
	R.ETFQNVFR.A	520.7616	911.4734<br>810.4257<br>663.3573<br>535.2987	12.5
	R.QYLDNPR.E	453.2274	614.3257<br>501.2416<br>386.2146<br>292.1292	9.7
A0A4Y7KLU8	R.VQVVGNAGR.Q	450.2565	672.3787<br>573.3103<br>474.2419<br>327.2027	9.3
	R.EETLILTPGLR.S	621.3586	769.4930<br>656.4090<br>543.3249<br>442.2772	13.5
	R.HIIQPR.G	382.2323	626.3984<br>513.3144<br>400.2303<br>492.2929	8.7
